# Location of births by health facility type: a time trend analysis from 1995 to 2023 in 130 low- and middle-income countries

**DOI:** 10.1016/j.eclinm.2025.103721

**Published:** 2025-12-30

**Authors:** Anna Gage, Madeleine Conrad, Chiara Sumich, Wes Warriner, Aduragbemi Banke-Thomas, Corinne Bintz, Kelly Bienhoff, Jessica Bishai, Rakhi Dandona, Mae Ashworth Dirac, Thomas Glucksman, Simon I. Hay, Megan Knight, Rafael Lozano, Ali H. Mokdad, Abdu Mohiddin, Bancy Ngatia, Marie-Jeanne Offosse, Jamilu Tukur, Marleen Temmerman, Bridget Stollfus, Asnake Worku, Enis Barış, Nicholas J. Kassebaum, Annie Haakenstad

**Affiliations:** a Institute for Health Metrics and Evaluation, University of Washington, Seattle, WA, USA; b Thinkwell Burkina Faso, Ouagadougou; c Public Health Foundation of India, New Delhi, India; d The National Data Management Center for Health, Ethiopian Public Health Institute, Addis Ababa, Ethiopia; e Centre of Excellence in Women and Child Health, Aga Khan University, Nairobi, Kenya; f Centre for Maternal Adolescent Reproductive and Child Health, London School of Hygiene & Tropical Medicine, London, United Kingdom; g Department of Anaesthesiology & Pain Medicine, University of Washington, Seattle, WA, USA; h Department of Global Health, University of Washington, Seattle, WA, USA; i Department of Health Metrics Sciences, University of Washington, Seattle, WA, USA; j Department of Family Medicine, University of Washington, Seattle, WA, USA; k School of Medicine, National Autonomous University of Mexico, Mexico City, Mexico; l Umaru Musa Yar'adua University, Federal Teaching Hospital Katsina, Katsina, Nigeria

**Keywords:** Maternal health, Neonatal health, Facility delivery, Health systems, Health care facilities, Low- and middle-income countries

## Abstract

**Background:**

With major increases in facility births in low- and middle-income countries (LMICs) since 1995, a key question is what types of facilities met the increased demand. Understanding the evolving delivery landscape is crucial to informing debates about optimal models for balancing access, quality, and equity. We studied the distribution of delivery location by health facility level and sector (private versus public) in 130 LMICs from 1995 to 2023.

**Methods:**

We used 745 data sources with delivery location information in our analysis. We first categorised births as in-facility or not, then further classified facility births by level (hospital or lower-level) and sector (public or private). We used spatiotemporal Gaussian process regression to model the share of births by facility type from 1995 to 2023 and compared delivery patterns to development and health indicators.

**Findings:**

In 2023, 47.5% (95% uncertainty interval [UI] 46.4–48.6) of deliveries in LMICs were in public hospitals, 19.2% (18.3–20.2) were in private hospitals, 13.0% (12.3–13.8) were in lower-level public facilities, 2.0% (1.9–2.2) were in private lower-level facilities, and the remaining 18.2% (17.3–19.2) were outside health facilities. In 106 countries, more than half of deliveries were in public hospitals, while in 12 countries, public lower-level facilities provided care for more than half of births. In only two countries were private hospitals used for more than half of births, while in the remaining ten countries no facility type provided the majority of care. Between 1995 and 2023, nearly two-thirds (62.1%) of the 41.0 percentage-point increase in facility births was borne by public hospitals. Delivery in lower-level facilities was more common in countries with lower levels of development and higher neonatal mortality rates.

**Interpretation:**

The mix of delivery locations represents the diversity of health systems worldwide. Our analysis highlights the pivotal and growing role of public hospitals in delivery care, though public lower-level delivery care is common in high-mortality contexts. Policy makers should account for the facility mix and the complex roles of public and private sectors when designing strategies to improve maternal and perinatal outcomes.

**Funding:**

The Gates Foundation.

**Research in context**

**Evidence before this study**

We conducted a title and abstract search of the literature in PubMed on May 14, 2025, using the search terms “in-facility delivery”, “facility delivery”, “place of delivery”, “institutional delivery”, and “public”, “private” or “hospital”. We additionally reviewed global health data repositories including the World Health Organization's Global Health Observatory, the World Bank World Development Indicators, and Countdown to 2030. The databases and hundreds of articles report on the percent of births that take place in a health facility versus those that take place outside a health facility. We identified 13 multi-country studies that disaggregate the type of health facility for delivery. Of these studies, four reported on the level of facility (hospital versus lower level), six reported on the sector (public versus private), and three reported on both level and sector. The studies note several geographic patterns in delivery mix, including higher utilisation of public facilities in sub-Saharan Africa and higher utilisation of private facilities in south and east Asia. We did not identify any studies that systematically estimate delivery location by type of facility for all low- and middle-income countries or examine trends in delivery location over time.

**Added value of this study**

In this study, we estimate, for the first time, delivery location by type of health facility for low- and middle-income (LMICs) countries from 1995 to 2023. Comprehensive estimates of deliveries by type of health facility are critical to understanding the diversity of ways health systems organise themselves around delivery care, which can provide insights about what care women and babies receive. These estimates are essential for designing and targeting interventions for where births are occurring and thereby making investments as effective as possible for improving maternal and perinatal health.

**Implications of all the available evidence**

Health systems approach delivery care with a variety of different facility types. Even so, across LMICs, public hospitals stand out as receiving the largest share of increased demand for facility births since 1995. Countries with the highest maternal and perinatal mortality rates rely heavily on lower-level facilities to conduct deliveries, which do not have capabilities to perform caesarean deliveries or blood transfusion in cases of emergency. Systems that rely on the private sector for delivery care should be carefully examined for concerns related to patient costs and inequities in access. Systems that rely primarily on the public sector should be assessed as to whether delivery care is vulnerable to inefficiency or low quality care.

## Introduction

Both the Millennium Development Goals and the Sustainable Development Goals emphasised increasing facility births as a key strategy for improving maternal and newborn health, a strategy particularly pertinent in low- and middle-income countries (LMICs) where facility delivery rates have historically been low.^1^ As compared to home births, delivering in a facility can better ensure mothers and their babies receive the health care they need to avoid disability and death resulting from complications of pregnancy and childbirth. In 2023, 82.4% of deliveries occurred in a health facility in LMICs, compared with 43.4% in 1995, marking a major expansion in facility deliveries around the world.^2^

How health systems achieved higher facility birth rates have likely differed substantially around the world, as a diversity of facilities provide delivery care. Prior literature has noted several distinct regional patterns in delivery location, for example, higher utilisation of public-sector health facilities in sub-Saharan Africa in comparison to greater use of private-sector facilities in south Asia and southeast Asia.^3^, ^4^, ^5^ Similarly, a greater proportion of births occur in hospitals rather than lower-level health facilities, such as clinics and primary health centres, in south Asia than in sub-Saharan African countries.^6^

A country's mix of delivery locations reflects the interplay of its health system investments and policies, the population's geographical and financial access to care, and women's preferences. Several current policy debates focus on where deliveries should occur to best increase facility births and improve health outcomes. For example, some have argued that relying only on the public sector is impractical and limits women's choice, while others contend that encouraging a large private sector exacerbates ineqities in care.^7^, ^8^, ^9^ Similarly, proposals to shift deliveries from lower-level facilities into hospitals aim to improve quality of care, but critics warn that doing so may reduce access, especially in rural or underserved areas.^10^, ^11^, ^12^

Despite the importance of these debates, there is little comprehensive evidence on what types of health facilities are providing delivery care and how this distribution has evolved over time. No studies to date have systematically assessed which facilities met the influx of demand for delivery care as facility delivery surged in LMICs. This study addresses the gap by estimating facility delivery trends across 130 LMICs from 1995 to 2023, disaggregated by facility ownership (public and non-profit versus private for-profit) and facility level (hospital versus lower-level facilities). With these estimates, policy makers can better understand which facilities met the increased demand for facility delivery, explore alternative models for delivery location mix, and identify the most frequently used facility types in order to target for interventions appropriately to reduce maternal and perinatal deaths.

## Methods

### Overview

We identified 745 population representative surveys and administrative sources with data on women's delivery locations and categorised facility births into public sector versus private sector and hospital versus lower-level facility. We used spatiotemporal Gaussian process regressions to model the distribution of deliveries by facility type for 130 LMICs from 1995 to 2023.

### Data seeking and extraction

To identify data sources for this analysis, we conducted a review of 2177 data sources linked to facility births using keywords used in modelling in-facility delivery as part of the Global Burden of Diseases, Injuries, and Risk Factors Study (GBD). Sources were identified from the Global Health Data Exchange, a large directory of surveys, vital registration, and other health data maintained by the Institute for Health Metrics and Evaluation. Included data sources must have asked about or reported delivery locations with options for different types of health facilities. We excluded data sources that were not nationally or regionally representative of women of reproductive age and sources collected before 1995. We chose 1995 as the baseline year given a relative lack of earlier data sources. While we use the term “pregnant women” throughout to align with the current literature and data sources, we acknowledge that not all pregnant people identify as female.

Sources in our analysis included survey series such as the Demographic and Health Surveys, Multiple Indicator Cluster Surveys, and Living Standards Measurement Study surveys as well as country-specific surveys and two administrative sources: vital registration systems in Mexico and Brazil. We extracted the location of delivery, year of delivery, and whether the delivery was caesarean from each identified data source. A total of 745 sources and 1312 location-years of data from 119 countries across all modelled regions were used to model delivery location, which recorded location for a total of 94 million births. Maps of the input data are in the Supplementary Material pp. 6–8.

### Data processing

We first categorised delivery location response options across all included data sources into the following groups: unknown (e.g., no response), non-facility (e.g., home, enroute), or health facility (e.g., government hospital, clinic). Health facility responses were then further classified into three mutually exclusive level categories (hospital, lower-level facility, or unknown health facility) and four mutually exclusive sector categories (public, private for-profit, private non-profit, or unknown).

While there is substantial diversity in what constitutes a hospital across countries, we defined a “hospital” as a facility that provides comprehensive emergency obstetric and neonatal care (CEmONC), with capabilities to provide caesarean deliveries (c-sections) and blood transfusions.^13^ Lower-level facilities encompass any other health facility performing deliveries without CEmONC capabilities; in different contexts these might include clinics, free-standing birthing centres, or primary health centres, with or without basic emergency obstetric and neonatal care (BEmONC) capabilities. We define sector based on facility ownership.^7^

Given the wide variety of response options across the data sources, we developed a systematic approach to categorise the options as consistently and accurately as possible. First, we reviewed the response option name and categorised it using common facility names listed in Supplementary Table S3. If we could not categorise the option using the name, we then reviewed the literature on the country's health system, for example from the Health Systems in Transition Series, Service Availability and Readiness Assessments, or ministry of health documents. Third, in cases where we could still not categorise the level, we assessed caesarean delivery rates as a sorting factor, a previously used approach for facility categorisation.^6^ Fourth, we compared the categorisation of responses across surveys from the same location and adjusted categorisations to ensure consistency across surveys. Finally, we consulted with experts on health systems across all regions to review the data sources, the categorisation of facility types according to the standard definitions we have articulated, and final estimates of delivery location over time. Additional details on the categorisation process are in the Supplementary Material (pp. 9–11). After categorising the responses, an average of 2% of women delivered in a facility with an unknown level and 7% delivered in facility with an unknown sector.

Using survey data, we calculated the proportion of facility births that took place in each combination of level and sector location by birth year for all children up to age 5 at the time of the survey, allowing us to estimate annual distributions of delivery location for up to five years preceding interviews. In administrative sources, we used all births in a given year. The share of births in private non-profit facilities was small in nearly all settings (<2% of all births). As such, given similarities in cost and quality with the public sector,^14^ we combined the private non-profit sector with the public sector for modelling.

### Modelling

We modelled four level- and sector-specific indicators on delivery location, each as a percentage of live births: 1) public or non-profit hospital, 2) public or non-profit lower-level facility, 3) private for-profit hospital, and 4) private for-profit lower-level facility. We additionally modelled two level-only indicators: percentage of live births delivered in a hospital and lower-level facility, respectively, regardless of sector.

Response options had differing levels of detail; some had information on both level and sector, while others had only one or the other. We therefore had nested criteria for inclusion in modelling. Location-years in which at least 85% of facility births had information on both level and sector were included in the level- and sector-specific models. Location-years where at least 95% of facility births had information on at least facility level were included in the level-only models.

We modelled the six delivery facility type indicators for all locations and years 1995–2023 using spatiotemporal Gaussian process regression (ST-GPR), a method used extensively in the GBD that estimates flexible time trends for quantities with high data densities, such as the delivery location quantities of focus here, by borrowing strength across space and time.^15^ ST-GPR is a three-step process which leverages data from surrounding geographies and time periods to produce estimates for a complete set of years and locations. First, we considered an array of covariates linked with delivery location and ran linear mixed-effects models for each combination of the covariates and our input data (full list of covariates in Supplementary Table S4). To make our results robust to the choice of covariates, we created an ensemble prediction of delivery location by the covariates, with weights based on the inverse out-of-sample root-mean-squared error of each model. In the second stage, we smoothed the residuals between the prediction and the delivery location input data using space and time weights, producing an updated time series for each location. Finally, a Gaussian process regression was used to estimate a final time series with uncertainty by incorporating input data variance and the differences between stage 1 (ensemble) and stage 2 (smoothed) estimates. The Supplementary Material (pp. 12–20) has additional information on the ST-GPR modelling process.

After modelling, we first summed the two level-specific outcomes and scaled the results to fit within the GBD total in-facility delivery envelope,^2^ then we summed the level- and sector-specific outcomes to fit within the hospital and lower-level envelopes, respectively. Finally, we aggregated the results into regions by weighing the results by the number of births in each location using birth estimates from the GBD.^16^ We report the modelled estimates for LMICs as defined by the World Bank 2025 income regions, excluding China due to lack of data.^17^

To understand patterns of delivery location across countries, we used Bayesian meta-regressions (MR-BRT) to compare our results to the neonatal mortality rate (NMR) and the Socio-demographic Index (SDI), a composite index of development calculated using educational attainment among the population aged 15 and older, the fertility rate for women under age 25, and ten-year lag-distributed income per capita.^18^ Further details on the MR-BRT models, a comparison to the maternal mortality ratio, and comparisons stratified by region are in the Supplementary Material (pp. 21, 37).

### Uncertainty

Sampling variance of the delivery proportion was estimated from each data source. In the third stage of the ST-GPR model, we sampled 1000 random draws of distributions defined by the mean, variance and a covariance function of the predicted delivery location distribution as described in the Supplementary Material for each location and year (Supplementary Material pp. 12–13). All subsequent scaling and aggregation was conducted on the 1000 draws, then 95% uncertainty intervals were calculated by taking the 2.5th and 97.5th percentile values.

### Ethics

Because this study employs only secondary, de-identified data, ethical approval was not required for this study.

### Role of the funding source

The study funder had no role in study design, data analysis, data interpretation, or writing.

## Results

Across the 130 LMICs in our study, 47.5% (95% UI 46.4–48.6) of births in 2023 took place in a public or non-profit hospital, 19.2% (18.3–20.2) occurred in a private for-profit hospital, and 13.0% (12.3–13.8) took place in a lower-level public or non-profit facility (Table 1). At 2.0% (1.9–2.2) of all births, very few births occurred in a private for-profit lower-level facility in 2023. The remaining 18.2% (17.3–19.2) of births across our study countries occurred outside of a health facility.

**Table 1:** Delivery location by facility type in 130 low- and middle-income countries, 2023

Location	Public and private non-profit hospital	Private for-profit hospital	Public and private non-profit lower-level	Private for-profit lower-level	Non-facility
**All study countries**	**47.5 (46.4–48.6)**	**19.2 (18.3–20.2)**	**13.0 (12.3–13.8)**	**2.0 (1.9–2.2)**	**18.2 (17.3–19.2)**
**Central Europe, Eastern Europe, and Central Asia**	**96 (95.3–96.6)**	**0.7 (0.5–1)**	**2.0 (1.7–2.4)**	**0.2 (0.1–0.3)**	**1.1 (0.8–1.5)**
Albania	98.5 (97.7–99)	0.6 (0.4–1)	0.0 (0–0)	0.1 (0–0.1)	0.8 (0.4–1.5)
Armenia	41.7 (36.2–48.5)	2.1 (1.2–3.3)	56.2 (49–61.6)	0.1 (0.1–0.1)	0.0 (0–0)
Azerbaijan	91.8 (87.9–94.7)	1.1 (0.7–1.8)	2.6 (1.2–4.6)	1.6 (0.7–3)	2.9 (1.3–5.5)
Belarus	99.8 (99.7–99.9)	0.0 (0–0)	0.1 (0–0.1)	0.1 (0–0.2)	0.0 (0–0)
Bosnia and Herzegovina	91.1 (85.1–95.3)	0.0 (0–0)	8.5 (4.4–14.3)	0.2 (0.1–0.4)	0.2 (0.1–0.4)
Georgia	92.8 (90–95)	4.1 (2.4–6.6)	0.6 (0.2–1.2)	2.2 (1–4)	0.3 (0.2–0.6)
Kazakhstan	99.8 (99.7–99.9)	0.0 (0–0)	0.0 (0–0.1)	0.1 (0–0.1)	0.0 (0–0.1)
Kyrgyzstan	98.7 (98.2–99.1)	0.7 (0.4–1)	0.1 (0.1–0.2)	0.3 (0.1–0.5)	0.2 (0.1–0.4)
Mongolia	87.9 (81.8–92.9)	0.5 (0.3–0.7)	11.1 (6.2–17.3)	0.1 (0.1–0.3)	0.3 (0.2–0.5)
Montenegro	99.3 (98.8–99.6)	0.0 (0–0)	0.4 (0.2–0.8)	0.1 (0.1–0.3)	0.1 (0.1–0.2)
North Macedonia	81.1 (73.1–87.9)	1.9 (1.1–3.1)	16.6 (10–24.5)	0.3 (0.1–0.5)	0.1 (0.1–0.2)
Republic of Moldova	98.8 (98.1–99.3)	0.1 (0–0.1)	0.4 (0.2–0.7)	0.4 (0.2–0.8)	0.3 (0.1–0.7)
Serbia	98.6 (97.6–99.2)	0.2 (0.1–0.4)	1.1 (0.6–2)	0.1 (0–0.2)	0.0 (0–0)
Tajikistan	91.1 (86.9–94.4)	0.8 (0.4–1.2)	0.9 (0.4–1.7)	0 (0–0.1)	7.1 (3.9–11.4)
Turkmenistan	91.6 (88–94.2)	0.1 (0.1–0.2)	8.2 (5.6–11.8)	0.1 (0–0.1)	0.0 (0–0)
Ukraine	99.6 (99.3–99.7)	0.2 (0.1–0.3)	0.2 (0.1–0.4)	0 (0–0)	0.0 (0–0)
Uzbekistan	98.5 (97.8–99)	1.0 (0.6–1.7)	0.1 (0.1–0.3)	0.1 (0–0.1)	0.3 (0.2–0.5)
**Latin America and Caribbean**	**65.1 (63.4**–**66.6)**	**21.0 (19.7**–**22.4)**	**6.1 (5.3**–**7)**	**2.0 (1.7**–**2.4)**	**5.8 (4.9**–**7)**
Argentina	64.9 (60.7–69.2)	32.1 (27.8–36.5)	2.6 (1.6–4)	0.1 (0.1–0.2)	0.2 (0.1–0.4)
Belize	82.7 (77.2–87.3)	8.4 (5.1–12.3)	0.1 (0–0.3)	4.9 (2.5–8.3)	3.8 (1.9–6.7)
Bolivia (Plurinational State of)	65.3 (55.9–73.6)	14.4 (9.2–20.4)	8.2 (4.5–13.5)	0.9 (0.4–1.9)	11.2 (5.7–19.8)
Brazil	72.1 (68.6–75.5)	26.7 (23.2–30)	0.8 (0.7–0.9)	0 (0–0)	0.4 (0.2–0.8)
Colombia	70.5 (63.8–77.1)	25.3 (18.5–31.7)	2.7 (1.3–5.1)	0.1 (0.1–0.3)	1.4 (0.7–2.5)
Costa Rica	96.9 (95.8–97.8)	2.1 (1.3–3.1)	0.6 (0.3–1.1)	0.2 (0.1–0.3)	0.2 (0.1–0.4)
Cuba	79.7 (73.3–86)	20.1 (14–26.6)	0 (0–0)	0 (0–0.1)	0.1 (0–0.1)
Dominica	85.8 (80.3–90.6)	13.2 (8.5–18.6)	0.2 (0.1–0.4)	0.3 (0.2–0.7)	0.5 (0.2–0.9)
Dominican Republic	79.9 (73–85.9)	18.9 (12.9–25.7)	0 (0–0.1)	0.1 (0–0.2)	1 (0.6–1.7)
Ecuador	60.4 (53–68)	34.2 (26.7–40.8)	1.4 (0.6–2.7)	3.2 (1.5–5.9)	0.8 (0.6–1)
El Salvador	94.1 (91.8–96)	2.8 (1.8–4.2)	0.4 (0.2–0.8)	0.2 (0.1–0.3)	2.6 (1.2–4.6)
Grenada	87.7 (82.5–91.9)	11.3 (7.1–16.3)	0.3 (0.1–0.6)	0.3 (0.1–0.6)	0.4 (0.2–0.7)
Guatemala	56.2 (52.2–60.1)	8.7 (5.8–12.1)	8.6 (7–10.2)	1.2 (0.7–1.9)	25.4 (22.8–28.1)
Haiti	29.6 (19–41.3)	4 (2.1–6.6)	9.2 (5.1–14.6)	0.8 (0.4–1.6)	56.3 (41.6–70.4)
Honduras	81.5 (76.4–85.6)	1.4 (0.9–2)	3.3 (1.7–5.5)	6.9 (4.2–10.4)	6.9 (4.3–10.5)
Jamaica	90.3 (86.4–93.6)	7.3 (4.5–11)	0.1 (0–0.2)	0.2 (0.1–0.3)	2.1 (1–4)
Mexico	50.8 (47–54.5)	22.5 (20.6–24.5)	16.2 (12.3–20.5)	4.1 (2.7–6)	6.3 (4.9–8.2)
Nicaragua	84.7 (77.5–90.4)	1 (0.5–1.6)	0.9 (0.3–1.9)	4.2 (2–7.6)	9.2 (4.5–15.9)
Paraguay	55.3 (46.8–63.5)	28.2 (21.2–35.5)	11.3 (6.4–17.2)	1.3 (0.6–2.6)	3.9 (2–6.9)
Peru	59.9 (53.2–66.5)	9.5 (7.2–12.4)	15.1 (10.4–20.3)	10 (6.6–14.2)	5.5 (3.6–8.1)
Saint Lucia	98.1 (97–98.9)	1.9 (1.1–3)	0 (0–0)	0 (0–0)	0 (0–0)
Saint Vincent and the Grenadines	89.7 (85.5–93.2)	8.9 (5.5–13.1)	0.4 (0.1–0.7)	0.3 (0.1–0.7)	0.7 (0.3–1.4)
Suriname	60.9 (54.5–67.1)	23.3 (18.4–28.9)	9.1 (5.5–13.5)	1.1 (0.5–2)	5.6 (3.2–8.8)
Venezuela (Bolivarian Republic of)	83.2 (77.4–88)	9.2 (5.7–13.7)	4 (1.9–7.2)	1.1 (0.4–2.1)	2.6 (1.2–4.9)
**North Africa and Middle East**	**54.5 (52.1**–**56.9)**	**22.3 (20.8**–**23.7)**	**6.2 (5.6**–**6.9)**	**0.9 (0.8**–**1)**	**16.2 (13.9**–**18.5)**
Algeria	82.2 (77.6–86.4)	6.3 (4.4–8.9)	10.5 (6.9–14.9)	0.2 (0.1–0.3)	0.8 (0.4–1.5)
Egypt	43 (37.9–49.3)	51.3 (45.2–56.4)	0.7 (0.3–1.3)	0.5 (0.2–1)	4.5 (2.3–7.7)
Iran (Islamic Republic of)	75.3 (68.1–82.1)	23.2 (16.3–30.3)	0.3 (0.1–0.6)	0.5 (0.2–0.9)	0.9 (0.4–1.6)
Iraq	77.7 (72.4–82.5)	14.6 (10.9–18.9)	0.1 (0–0.2)	0.1 (0.1–0.2)	7.5 (4.3–11.8)
Jordan	64 (58.7–69.9)	34.6 (28.7–40)	0 (0–0.1)	0.1 (0–0.1)	1.2 (0.9–1.6)
Lebanon	20.2 (17.6–22.9)	78.4 (75.7–80.9)	0 (0–0.1)	1.2 (0.6–2.3)	0.2 (0.1–0.5)
Libya	87.6 (82.7–91.6)	9.8 (6–14.5)	1.1 (0.5–2.3)	1.3 (0.5–2.4)	0.2 (0.1–0.3)
Morocco	73.8 (65.3–81.3)	4.5 (2.7–7.1)	16.1 (8.9–24.2)	0.2 (0.1–0.4)	5.4 (2.5–9.9)
Palestine	66.9 (62–71.7)	30.8 (25.7–35.5)	0.2 (0.1–0.3)	2.1 (1.2–3.3)	0.1 (0.1–0.2)
Syrian Arab Republic	60.8 (50.7–69.9)	19.4 (12.5–27.3)	0.4 (0.1–0.9)	5.9 (3–10)	13.6 (6.5–22.9)
Tunisia	82 (76.7–86.5)	17.4 (12.9–22.6)	0.1 (0–0.1)	0.1 (0–0.1)	0.5 (0.3–0.7)
Türkiye	55.5 (49.5–61.9)	35.4 (29.6–41.2)	6.4 (3.7–10)	0.7 (0.3–1.3)	2 (0.9–3.7)
Yemen	29.5 (26.7–32.4)	9.4 (8.1–10.9)	8.7 (7.2–10.4)	1.5 (1.1–1.9)	50.9 (46.6–55)
**South Asia**	**54.2 (52.9**–**55.6)**	**28.1 (26.9**–**29.4)**	**6.1 (5.8**–**6.6)**	**1.2 (1**–**1.5)**	**10.3 (10**–**10.8)**
Afghanistan	36.4 (34–38.9)	7.8 (6.9–9)	22.6 (20.7–24.7)	2.3 (1.9–2.7)	30.9 (27.8–34.1)
Bangladesh	31.5 (27.1–36.2)	15.7 (11.9–19.3)	6.4 (4.2–9)	17.9 (14–22)	28.5 (25.2–32.2)
Bhutan	57.3 (48.3–66.1)	16.2 (10.6–22.8)	12.9 (7.8–19.4)	1.6 (0.7–3)	12.1 (6.1–20.1)
India	58.2 (56.8–59.6)	27.1 (26–28.4)	6.8 (6.4–7.4)	0.1 (0.1–0.2)	7.7 (6.9–8.9)
Nepal	45.3 (41.8–48.6)	15.9 (13.8–18.2)	19.5 (17–22.1)	0.3 (0.2–0.4)	19.1 (15.9–22.7)
Pakistan	38.8 (33.6–44.5)	38.8 (33.6–44.2)	0.2 (0.1–0.4)	0 (0–0)	22.1 (15.5–29.8)
**Southeast Asia, East Asia, and Oceania**	**48.4 (45.9**–**51.2)**	**14.1 (11.6**–**16.8)**	**13.6 (11.8**–**15.6)**	**11.0 (8.6**–**13.5)**	**12.9 (11.2**–**15.3)**
Cambodia	35.4 (32.2–38.3)	5.7 (4.2–7.4)	46.7 (43.7–49.7)	9.8 (8.3–11.6)	2.5 (1.7–3.4)
Democratic People's Republic of Korea	76.4 (69.6–81.7)	10.3 (6.3–14.9)	6.5 (3.1–11.3)	0.6 (0.2–1.1)	6.3 (3.9–9.7)
Fiji	95.9 (94.6–97)	0 (0–0)	3.8 (2.8–5.2)	0.1 (0.1–0.2)	0.1 (0.1–0.2)
Indonesia	28.2 (22.7–35)	19.1 (14.2–25)	14.6 (10–19.5)	27.2 (21–33.8)	10.9 (6.5–16.7)
Kiribati	70.6 (63.6–76.6)	0 (0–0)	17.8 (13.3–22.6)	0.1 (0.1–0.2)	11.5 (6.6–18.3)
Lao People's Democratic Republic	57.7 (46.1–67.5)	0.2 (0.1–0.3)	11.6 (7.3–16.8)	1.6 (0.7–3)	28.9 (19–41)
Malaysia	80.7 (72.7–87.3)	4.5 (2.6–7)	11.4 (6.1–18.1)	2.9 (1.2–5.4)	0.6 (0.3–1.1)
Maldives	78.8 (73.3–84.1)	16.9 (12–22.5)	2.1 (1.1–3.5)	0.1 (0.1–0.3)	2.1 (1–3.7)
Marshall Islands	91.7 (87.2–94.9)	0.4 (0.2–0.6)	4.7 (2.3–8.5)	0.3 (0.1–0.7)	2.9 (1.4–5.3)
Micronesia (Federated States of)	83.7 (75.1–89.8)	0.2 (0.1–0.3)	4.5 (2.1–8)	0.3 (0.1–0.7)	11.4 (5.6–20.4)
Myanmar	54.9 (42.8–67.2)	8.7 (5.2–13.1)	3.5 (1.7–6.3)	0.2 (0.1–0.4)	32.7 (19.5–46.7)
Papua New Guinea	33.4 (24.2–43.8)	1.1 (0.6–1.8)	27.4 (18.9–36.3)	0.1 (0–0.1)	38 (24.9–53.2)
Philippines	40.9 (37–45)	24.6 (21.2–27.8)	16 (12.8–19.6)	0.2 (0.1–0.2)	18.4 (14.9–22.5)
Samoa	79.6 (74–84.4)	0 (0–0)	9.9 (7.1–13.4)	0.1 (0.1–0.2)	10.4 (6.3–16.1)
Solomon Islands	83.6 (75.5–89.7)	0.2 (0.1–0.3)	8.3 (4.1–14.3)	0.2 (0.1–0.5)	7.7 (3.7–13.5)
Sri Lanka	98.4 (97.5–99)	0.3 (0.2–0.5)	0 (0–0)	1.1 (0.6–1.9)	0.2 (0.1–0.3)
Thailand	91.3 (89.3–93.1)	7.9 (6.1–9.9)	0.1 (0–0.2)	0.3 (0.2–0.5)	0.3 (0.2–0.5)
Timor-Leste	38.2 (26.9–49.6)	0.7 (0.4–1.2)	19.3 (12–27.7)	0.3 (0.1–0.5)	41.5 (27.6–56.5)
Tonga	97.4 (96–98.2)	0.7 (0.4–1.1)	0 (0–0.1)	0.7 (0.4–1.2)	1.2 (0.6–2.3)
Vanuatu	83.3 (75.7–89.4)	0.2 (0.1–0.3)	6.7 (3.3–11.8)	0.2 (0.1–0.5)	9.5 (4.7–16.4)
Viet Nam	79.4 (74.2–84.2)	5 (3.5–6.9)	13.2 (8.8–18.7)	0.3 (0.2–0.5)	2.1 (1.3–3.4)
**Sub-Saharan Africa**	**32.6 (30.7**–**34.5)**	**10.8 (9.4**–**12.2)**	**23.8 (22**–**25.7)**	**1.1 (0.9**–**1.5)**	**31.7 (29.9**–**33.8)**
Angola	41 (30.6–52)	1.1 (0.6–1.9)	24.1 (15.9–33)	0.2 (0.1–0.4)	33.5 (20.7–48.1)
Benin	46.9 (43.5–50.4)	7.4 (6–9.1)	38.4 (34.7–42)	0.1 (0.1–0.1)	7.2 (5.5–9.3)
Botswana	84.6 (77.4–89.9)	4.1 (2.4–6.3)	10.8 (5.7–17.8)	0.4 (0.2–0.9)	0.2 (0.1–0.3)
Burkina Faso	9.7 (8.6–10.9)	0.3 (0.2–0.5)	83.9 (81.7–85.8)	0.3 (0.3–0.4)	5.8 (4.2–7.8)
Burundi	35.4 (28.5–43.6)	1.5 (0.9–2.4)	45.4 (36.9–52.8)	3.5 (2.3–5.1)	14.2 (8.3–22.8)
Cabo Verde	66.9 (58–74.6)	12.9 (8.2–18.6)	13.7 (7.5–21.7)	0.2 (0.1–0.5)	6.2 (2.8–11.5)
Cameroon	39.1 (31.3–47.8)	5.9 (3.5–9.2)	30.5 (22.4–38.1)	0.1 (0.1–0.2)	24.3 (16.8–33.1)
Central African Republic	34.1 (26.1–42.8)	3 (1.9–4.6)	26.8 (19.4–34.4)	1.2 (0.7–1.7)	34.9 (24.2–47.1)
Chad	19.5 (14–26.4)	1.4 (0.8–2.3)	8.7 (5.3–12.9)	0.2 (0.1–0.4)	70.2 (60.7–78.3)
Comoros	75.3 (72.3–78.2)	3.9 (3–5)	16.4 (13.8–19.3)	0.1 (0.1–0.2)	4.3 (3.1–5.6)
Congo	66.1 (59.8–72)	3.6 (2.3–5.3)	15.1 (9.2–21.9)	10.1 (8.5–12.1)	5.1 (2.4–9.1)
Côte d'Ivoire	43.6 (39.4–47.7)	3.4 (2.4–4.5)	34.7 (30.5–38.7)	0.8 (0.6–1)	17.6 (14–21.9)
Democratic Republic of the Congo	33.7 (25.7–42.6)	18.3 (12.5–25.3)	25.1 (17.9–32.8)	10.9 (7.2–15.8)	12 (7.4–18)
Djibouti	89.9 (85.2–93.5)	1.7 (1–2.8)	3.3 (1.6–6)	0.2 (0.1–0.4)	4.8 (2.3–9.3)
Equatorial Guinea	60.1 (51.1–68.3)	13.7 (8.8–19.3)	11.2 (6.1–17.9)	1.6 (0.7–3.2)	13.3 (7.2–21.4)
Eritrea	51.3 (35.5–65.7)	0.4 (0.2–0.7)	5.7 (2.7–10.3)	0.1 (0–0.3)	42.5 (26.7–59.5)
Eswatini	83.1 (79.4–86.3)	2.4 (1.5–3.5)	7.3 (5.4–9.7)	0.5 (0.3–0.9)	6.7 (4.5–9.4)
Gabon	67.4 (59.7–74.8)	22.3 (15.8–29.2)	2.5 (1.1–4.6)	4.8 (2.3–8.5)	3 (1.4–5.5)
Gambia	32.4 (27.5–37.5)	6.6 (4.6–9.1)	49 (43.4–54.7)	0.2 (0.2–0.3)	11.8 (7.5–17.1)
Ghana	57.3 (54–60.3)	9.6 (7.9–11.5)	17.8 (15.6–20.1)	0.6 (0.5–0.8)	14.7 (12.1–17.5)
Guinea	21.4 (15.7–27.3)	5.6 (3.5–8.3)	37.2 (28.7–45.4)	0.3 (0.2–0.4)	35.6 (24.8–47.6)
Guinea-Bissau	49.2 (37.2–60.5)	0.7 (0.4–1.2)	11.7 (6.5–18.6)	0.4 (0.2–0.7)	38 (26.4–52.1)
Kenya	48.2 (44.5–51.4)	14.5 (12.8–16.4)	18.6 (16.7–20.4)	0.6 (0.5–0.7)	18.1 (14.3–23.6)
Lesotho	68.6 (62.1–74.4)	4.7 (3–7)	17.3 (11.7–23.7)	0.2 (0.1–0.4)	9.2 (7.2–11.4)
Liberia	34.9 (28.9–41.3)	15.8 (11.8–20.3)	35.7 (29–41.9)	0.1 (0.1–0.2)	13.5 (8.8–19.4)
Madagascar	12.5 (10.1–15)	4.8 (3.7–6.1)	24 (20.2–28.3)	0.1 (0.1–0.1)	58.5 (52.9–64.3)
Malawi	59.4 (53.8–65.5)	3.3 (2.3–4.7)	34.5 (28.2–40.4)	0.3 (0.2–0.4)	2.5 (1.5–3.8)
Mali	7.5 (5.5–9.8)	3.1 (2–4.5)	62.6 (53.2–70.2)	1.3 (0.9–1.8)	25.5 (17–36.1)
Mauritania	46 (39.4–52.7)	2.7 (1.8–4)	30.5 (24.4–36.5)	0.1 (0.1–0.2)	20.7 (14.7–28.5)
Mauritius	79.4 (72.3–85.3)	14.8 (9.5–21.1)	4.3 (2.1–7.7)	1.4 (0.5–2.8)	0 (0–0)
Mozambique	35.8 (27.9–44.7)	0.4 (0.2–0.6)	28.3 (19.5–36.3)	0.2 (0.1–0.3)	35.4 (30.8–40.3)
Namibia	83.7 (78.4–88)	7.7 (4.8–11.4)	2.5 (1.2–4.3)	0.2 (0.1–0.4)	5.9 (3.3–9.7)
Niger	7.4 (3.8–12.3)	0.8 (0.3–1.5)	30.9 (19.3–44.8)	0.4 (0.2–0.8)	60.6 (43.7–75.2)
Nigeria	26.4 (22.5–30.6)	16 (13.2–18.9)	12 (8.1–16)	0.3 (0.2–0.5)	45.3 (43.2–47.5)
Rwanda	45.5 (40.7–50.8)	1.7 (1.1–2.5)	48.3 (42.7–53.4)	0.2 (0.1–0.2)	4.3 (2.7–6.4)
Sao Tome and Principe	92.6 (89.4–94.9)	0 (0–0.1)	3.3 (1.8–5.5)	0.1 (0–0.2)	3.9 (2.2–6.4)
Senegal	23.6 (20.1–27.4)	6.7 (4.8–9.1)	61.9 (58.1–65.2)	0.1 (0.1–0.1)	7.7 (6.5–9)
Sierra Leone	33.5 (28.8–38.4)	2.9 (2–4)	49.9 (44.9–54.9)	0.3 (0.2–0.4)	13.4 (9.4–18.5)
Somalia	25.7 (15.6–37.4)	10.4 (5.3–16.5)	3.3 (1.5–6.5)	1.1 (0.4–2.5)	59.4 (42.6–75)
South Africa	85.4 (80.2–89.6)	3.8 (2.4–5.7)	7.9 (4.4–12.6)	0.1 (0–0.1)	2.8 (1.4–5)
South Sudan	6.7 (3.2–12.3)	0.6 (0.2–1.3)	3.9 (1.6–7.7)	0.1 (0–0.2)	88.6 (80.2–94.2)
Sudan	41 (26.6–57.1)	2.0 (1.1–3.3)	0.3 (0.1–0.6)	0.1 (0–0.2)	56.6 (40.1–71.7)
Togo	39.9 (31.9–48.9)	10 (6–14.9)	34.8 (25.8–43.1)	3 (1.8–4.5)	12.3 (7.4–19.4)
Tuvalu	95.9 (94.1–97.4)	3.9 (2.4–5.6)	0 (0–0.1)	0.1 (0.1–0.3)	0 (0–0.1)
Uganda	38.6 (31.5–46.6)	25.1 (18.9–31.4)	22.3 (15.7–29.4)	0.1 (0.1–0.2)	13.8 (8.3–20.8)
United Republic of Tanzania	56.8 (53.7–59.9)	1.5 (1.1–2)	23.4 (20.9–25.9)	0.8 (0.7–1.1)	17.4 (14.8–20.4)
Zambia	42.5 (36.4–49.4)	2.3 (1.4–3.5)	44.8 (38.3–50.7)	0.2 (0.1–0.2)	10.4 (6.3–15.9)
Zimbabwe	51.2 (45–57.7)	4.4 (2.9–6.4)	29.9 (23.7–36.2)	0.3 (0.2–0.5)	14.1 (9.9–19.4)

**Fig. 1:**
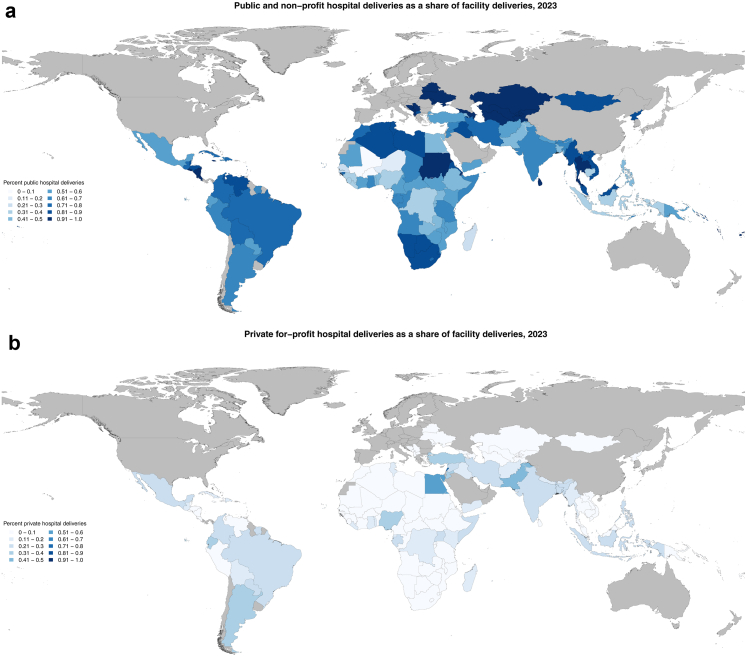

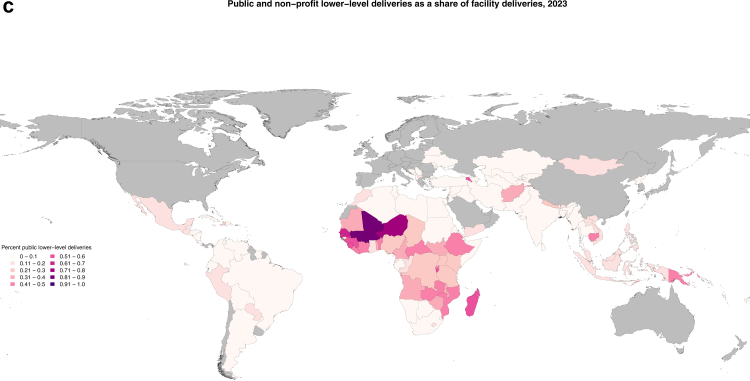
**Delivery location as a share of facility deliveries, 2023**. Fig. 1a: Public and non-profit hospital deliveries as a share of facility deliveries, 2023. Fig. 1b: Private hospital deliveries as a share of facility deliveries, 2023. Fig. 1c: Public and non-profit lower-level deliveries as a share of facility deliveries, 2023. Notes: Absolute numbers shown in Supplementary Table S5.

The mix of delivery locations varied substantially across LMICs in 2023 (Fig. 1). Public and non-profit (hereafter referred to as public) hospital births comprised a wide range of facility births, from less than 10% of facility births in Mali (Fig. 1a), where lower-level public facilities are more dominant (Fig. 1b), to more than 90% of facility births in 32 countries across all regions of the world. Public hospitals were the location of more than half of all births in 106 of the 130 countries in our study. Public lower-level facilities provided care for more than half of births in 12 countries, including 11 in west and east Africa, and Kyrgyzstan. In Burkina Faso, for example, 83.9% (95% UI 81.7–85.8) of facility births were in public lower-level facilities. Private for-profit (hereafter referred to as private) hospitals accounted for more than half of deliveries (Fig. 1b) in just two countries, Egypt and Lebanon. In the remaining ten countries, no single facility type provided the majority of care.

**Fig. 2:**
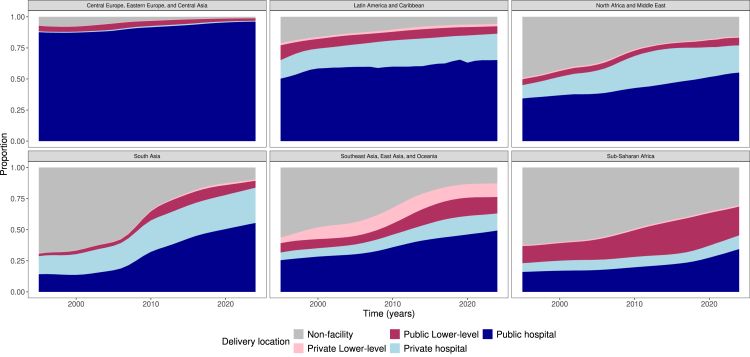
**Proportion of deliveries by delivery location by region, 1995–2023**.

**Fig. 3:**
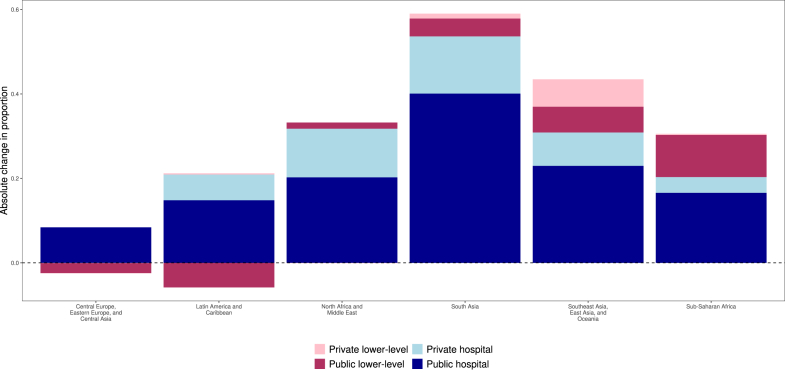
**Absolute change in facility delivery by facility type and region, 1995–2023**.

Between 1995 and 2023, the share of births that took place in a facility increased by 41.0 percentage points (95% UI 39.4–42.4) in study countries, with 62.1% (59.3–64.8) of the increase driven by more deliveries in public hospitals. However, the composition of deliveries by facility level and ownership, and trends therein over time, were distinct across regions (Fig. 2). In low- and middle-income Europe and central Asia, facility births remained high and public hospitals dominated throughout the period, with 96.0% (95.3–96.6) of all births occurring in public hospitals in 2023. Similarly, in Latin America and the Caribbean, public hospitals were the location of the majority of births in 2023 (65.1%, 63.4–66.6), although this region does have a more sizeable private sector, with 21.0% (19.7–22.4 of births taking place in private hospitals in 2023. This private-sector usage was driven in large part by private-sector deliveries in Argentina, Ecuador, and Brazil. Increases in use of both public and private hospitals for deliveries drove much of the increase in facility deliveries in Latin America and the Caribbean from 1995 to 2023, while there was a shift away from public lower-level facilities (Fig. 3).

In other regions, public hospitals were less dominant, although they still were the location of over half of facility deliveries in all regions on average in 2023. In south Asia and north Africa and the Middle East, private hospitals accounted for 28.1% (95% UI 26.9–29.4) and 22.6% (20.9–24.1), of births respectively. Distinct to sub-Saharan Africa was the sizeable portion of births that took place in public lower-level facilities, which was 23.8% (22.0–25.7) of total births and 32.5% (30.2–34.9) of facility births. Despite the larger diversity of birthing facility type, public hospitals nonetheless carried the largest share of increased demand for facility births from 1995 to 2023 in these regions as well as in southeast Asia, east Asia, and Oceania (Fig. 3). Private lower-level facilities accounted for small portions of the growth in facility births across most regions except for south Asia and southeast Asia, east Asia, and Oceania; this was driven primarily by private lower-level use in Bangladesh and Indonesia. Maps of the change over time by country are included in the Supplementary Material (pp. 34–35).

**Fig. 4:**
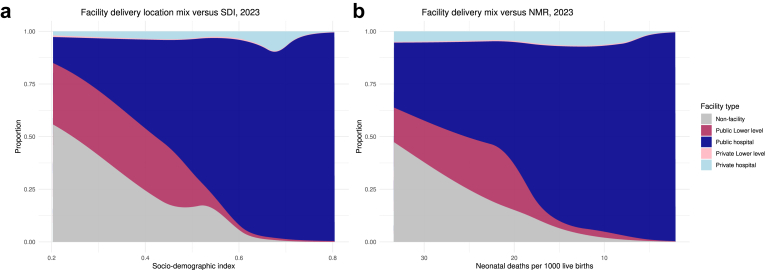
**Facility delivery location mix versus social and economic development and neonatal health, 2023**. Fig. 4: a. Facility delivery location mix versus SDI, 2023. Fig. 4b: Facility delivery location mix versus NMR, 2023. Notes: NMR: Neonatal mortality rate. SDI: Socio-demographic Index, or a composite indicator of total fertility rate among women under age 25, mean educational attainment among the population aged 15 and older, and ten-year lag-distributed income per capita, a measure of social and economic development. NMR has a reverse scale. NMR and SDI sourced from the Global Burden of Diseases, Risk Factors, and Injuries Study 2023.

We compared delivery location mix to key aspects of social and economic development and neonatal health. Fig. 4a depicts facility location mix against SDI in 2023. Countries with low SDI values have greater shares of births in public lower-level facilities in comparison to locations with high SDI values. Countries with high SDI predominantly rely on public hospitals for deliveries. Similarly, in countries with high neonatal mortality, public lower-level deliveries are more common, which decreases in favour of more public hospital usage in locations with low neonatal mortality (Fig. 4b), with high public lower-level use particularly in countries with over 18 neonatal deaths per 1000 live births. Private hospitals were used across countries with a wide range of SDI and NMR values.

## Discussion

The role of health facilities in childbirth has grown substantially since 1995. Our study emphasised the major role of public hospitals in increases in facility delivery in a wide range of LMICs, but also that this was not the case everywhere—private hospitals and public lower-level facilities are key delivery locations in select health systems. As such, our results denoted major differences in delivery location across world regions. Our findings can help inform current policy debates by identifying an array of different arrangements for providing maternal and perinatal health care.

Varying policies and patient preferences shaped the diversity of delivery location arrangements that we found. For example, in India, where we found public hospitals made up the majority of the increase in facility deliveries, the government implemented a large conditional cash transfer scheme in 2005 to encourage facility delivery.^19^ While women received incentives if they gave birth in a government or accredited private facility, few private providers joined the program and a minority of women were aware of the private facility options. As a result, most women attended public hospitals.^20^ Public hospitals similarly made up a majority of the increase in facility deliveries in Guatemala. There, the Ministry of Health focused on improving culturally appropriate care and improving facility capacity for delivery in public facilities to encourage underserved indigenous populations to deliver in health facilities.^21^ In contrast, in Egypt, the rise in private hospital deliveries in part reflects patient preferences, driven by perceptions that private facilities offer better privacy, communication, and respectful care compared to public hospitals.^22^

The predominant use of public hospitals for delivery does not imply that it is appropriate for all contexts. We found private hospitals were used more frequently in several countries in north Africa and the Middle East. Our results on the sector of facility used for delivery can support comparative investigations into equity and costs as barriers to facility births in countries with different patterns. Though not uniform across countries, public-sector facilities are often more affordable than private facilities.^3^^,^^5^^,^^23^ Government tax revenue or social insurance programmes tend to cover at least some service costs in public facilities. Eliminating patient fees in the public sector for maternal and child health care was also a policy commonly adopted in LMICs over the past 30 years.^24^ Where the private sector is more frequently used, deliveries may involve more substantial out-of-pocket costs.^25^ This is because, in most LMICs, prepaid private insurance is not common, nor are arrangements for governments to reimburse providers for private-sector care.^26^ These patient costs can result in inequities in access to care. Pakistan's increase in private hospital deliveries, for example, was driven primarily by upper wealth quintiles, while poorer women are more likely to use the public sector.^27^ The connection to inequities in access is one explanation for why private hospital use does not monotonically decline with SDI; countries in the middle of the income distribution tend to have more inequality but also have higher purchasing power to select the private sector.^28^

The dominant sector of the delivery facilities may also shape provider incentives, which in turn can impact quality of care. Public providers typically are paid by fixed salaries with no incentives for output or quality, which can result in inefficiency, low-quality care, and absenteeism, but may promote equitable service delivery.^29^ The fee-for-service arrangements common in private facilities are associated with greater efficiency and responsiveness, but may drive over-provision of care,^30^ such as non-medically indicated caesarean deliveries.^31^ Our findings offer a foundation for case studies that can further explore how delivery location shapes care quality through provider incentives.

We found that public lower-level facilities contributed substantially to the increase in facility deliveries in sub-Saharan Africa, especially in several west and central African countries. These facilities, often closer to populations, particularly in rural areas, help improve access to care.^11^^,^^32^^,^^33^ However, they typically have lower readiness to manage life-threatening obstetric complications than hospitals,^34^ though there is wide variation in facility capacity between and within countries.^35^ We found that lower-level facilities are a more dominant location for delivery care in countries with high neonatal and maternal mortality. Prior studies have also linked higher perinatal mortality to greater reliance on lower-level facilities.^6^^,^^36^^,^^37^ In Ethiopia for example, where we found deliveries in public lower-level facilities increased by 21.3% (95% UI 16.8–26.5), a prior study found that a major health centre construction programme increased births in lower-level facilities but did not reduce neonatal mortality.^38^ In contrast, we found that in Latin America and the Caribbean, a comparatively lower mortality region, deliveries shifted from public lower-level facilities to public hospitals over the study period. While our analysis is ecological, not causal, these cases can be carefully examined to inform the debate about the optimal mix of delivery locations, highlighting the need for thorough assessments of care quality by level and sector.

Our findings have several important policy implications. First, they suggest that efforts to improve maternal and perinatal outcomes must go beyond increasing facility deliveries and focus on strengthening the quality of care within the dominant delivery sectors and facility types in each context. For example, countries where lower-level public facilities are important sources of delivery care may need targeted investments in emergency obstetric readiness, while settings with high private-sector use may need regulatory approaches to ensure quality and manage costs. It will also be crucial to continue to monitor the impact of such policies on delivery location, caesarean delivery rates and health outcomes. Moving forward, donor reductions in countries with a high reliance on development assistance for health may constrain budgets in the public and non-profit sectors, imperilling future progress in facility deliveries or prompting greater private sector use. Second, the sectoral patterns we identify point to the importance of equity-focused policies. As highlighted by some of the examples from Pakistan and Guatemala above, the choice of delivery facility may differ for populations of different wealth, ethnicity or urbanicity. While we were limited to national analyses, further disaggregation of delivery location by these sub-groups will help to identify barriers to accessing high quality care such as cost, distance, or lack of culturally appropriate care and guide policies to close equity gaps. Third, our findings provide a roadmap for future research: country case studies can examine the policies and preferences that shaped the delivery mix as well as the implications of that mix for access, equity, quality, and health outcomes.

Finally, to improve measurement of delivery location, population surveys should offer response options that align with the health system's organisation, and more nationally representative health facility surveys are needed to assess readiness to provide comprehensive emergency maternity and newborn care.^39^ Relying solely on facility type can mislead policy decisions if it is assumed to reliably indicate access to emergency obstetric care, respectful care, or financial protection. Further evidence from facility surveys can provide much more nuance into a particular health system. Importantly, future measurement and monitoring of facility delivery is threatened by the recent cancellation of the Demographic and Health Surveys, which was a critical source for this analysis.^40^

To the best of our knowledge, this study is the first to systematically categorise delivery location by health facility level and sector and provide comparable estimates across LMICs and over time. Nevertheless, there are several limitations to note. First, as most of our data sources rely on self-report, there may be biases related to patients' lack of knowledge of the level or ownership of the facilities attended, or some patients may colloquially refer to all health facilities as a ‘hospital’. Surveys may also have excluded or simplified some facility types in the response options, leading to measurement error. Apart from Mexico and Brazil, we were unable to validate these responses with administrative data capturing the location of delivery, despite extensive review of administrative sources. We conducted extensive expert review to ensure our results were plausible, nonetheless, validation with non-survey sources is an area we hope to improve upon in future iterations of this work. Second, the lack of standardised facility definitions across countries also limits further disaggregation among lower-level health facilities, for example, between health centres and dispensaries. Similarly, due to small proportions of deliveries in the private non-profit sector, we grouped these with the public sector for modelling; however, disaggregating these types may be more relevant for some countries. Third, while we show ecological relationships between delivery location and mortality, the associations shown should not be interpreted causally. Fourth, we were unable to capture the role of intrapartum referrals in our analysis, as most data sources only contain information about the place of birth rather than any additional facilities visited during labour or immediately postpartum. Fifth, we were unable to identify data sources from eleven of the LMICs that were modelled. In future iterations of these estimates, we will re-examine the availability of data, including from routine data systems, to assess whether high-income countries or China could also be included.

Increases in facility births in LMICs in recent decades have largely been driven by increased use of public hospitals, with several notable exceptions. The diversity of delivery location mixes across LMICs underscores the importance of tailoring maternal and perinatal policies to context-specific needs. By systematically categorising delivery locations by facility level and sector, this research offers a foundation for future studies to explore the implications of delivery location patterns for health equity, quality, and outcomes, supporting efforts to improve maternal and perinatal care worldwide.

## Contributors

Managing the estimation process: Annie Haakenstad, Anna Gage, Nicholas Kassebaum.

Writing the first draft of the manuscript: Anna Gage, Annie Haakenstad.

Providing data or critical feedback on data sources: Wes Warriner, Jessica Bishai, Bridget Stollfus, Corinne Bintz, Megan Knight, Rafael Lozano.

Accessed and verified data; extracting, cleaning or cataloging data; designing or coding figures and tables: Madeleine Conrad, Chiara Sumich, Wes Warriner, Jessica Bishai, Bridget Stollfus, Thomas Glucksman.

Drafting the work or revising it critically for important intellectual content: Aduragbemi Banke-Thomas, Kelly Bienhoff, Rakhi Dandona, Mae Ashworth Dirac, Bancy Ngatia, Asnake Worku, Rafael Lozano, Ali Mokdad, Abdu Mohiddin, Marie-Jeanne Offosse, Jamilu Tukur, Marleen Temmerman, Enis Baris.

Managing the overall research enterprise: Annie Haakenstad, Nicholas Kassebaum, Kelly Bienhoff.

## Data sharing statement

Input data sources and code are available at https://github.com/ihmeuw/delivery_location. Estimates produced in these analyses are available from the Global Health Data Exchange website (ghdx.healthdata.org).

## Editor note

The Lancet Group takes a neutral position with respect to territorial claims in published maps and institutional affiliations.

## Declaration of interests

Anna Gage, Kelly Bienhoff, Nicholas Kassabaum report an institutional grant from the Gates Foundation. Nicholas Kassabaum reports consulting fees from Bristol Meyers Squib for travel expenses and fees for presentation of GBD results at international conference, unrelated to current work, and Fujifilm Sonosite and Philips Medical for product development consultation, unrelated to current work. Rakhi Dandona reports funding from USAID, NIHR (UK), Mariwala Foundation, University of Washington, Indian Council of Medical Research. Jamilu Tukur reports travel support from Bayero University to attend a scientific meeting of the West African College of Surgeons in Bamako, Mali.
