# Supplementary material for: Location of births by health facility type: a time trend analysis from 1995 to 2023 in 130 low- and middle-income countries
Source: eClinicalMedicine. 2025 Dec 30;91:103721. doi: 10.1016/j.eclinm.2025.103721 (PMC12804159; doi:10.1016/j.eclinm.2025.103721)
Supplement: Supplementary Figures and Tables [file mmc1.pdf]

Supplementary material to “Location of births by health facility type: a time trend analysis from 1995 to 2023 in 130 low- and middle-income countries”

Table of Contents

Part 1. Supplementary methods **2**
Data sources 2
Health facility categorization 5
Modelling strategy 7
Analysis 17

Part 2. Supplementary results **18**
Private for-profit lower-level deliveries, 2023 18
Private non-profit deliveries, 2023 18
Numbers of deliveries by location, 2023 19
Delivery location by country, 1995 24
Change in delivery location by country, 1995-2023 30
Delivery location mix versus sociodemographic index and neonatal mortality by region, 2023 31
Delivery location mix versus maternal health, 2023 33

References **34**

Part 1. Supplementary methods

Data sources

Supplementary Figure 1. Map of input data of delivery in a hospital of any sector

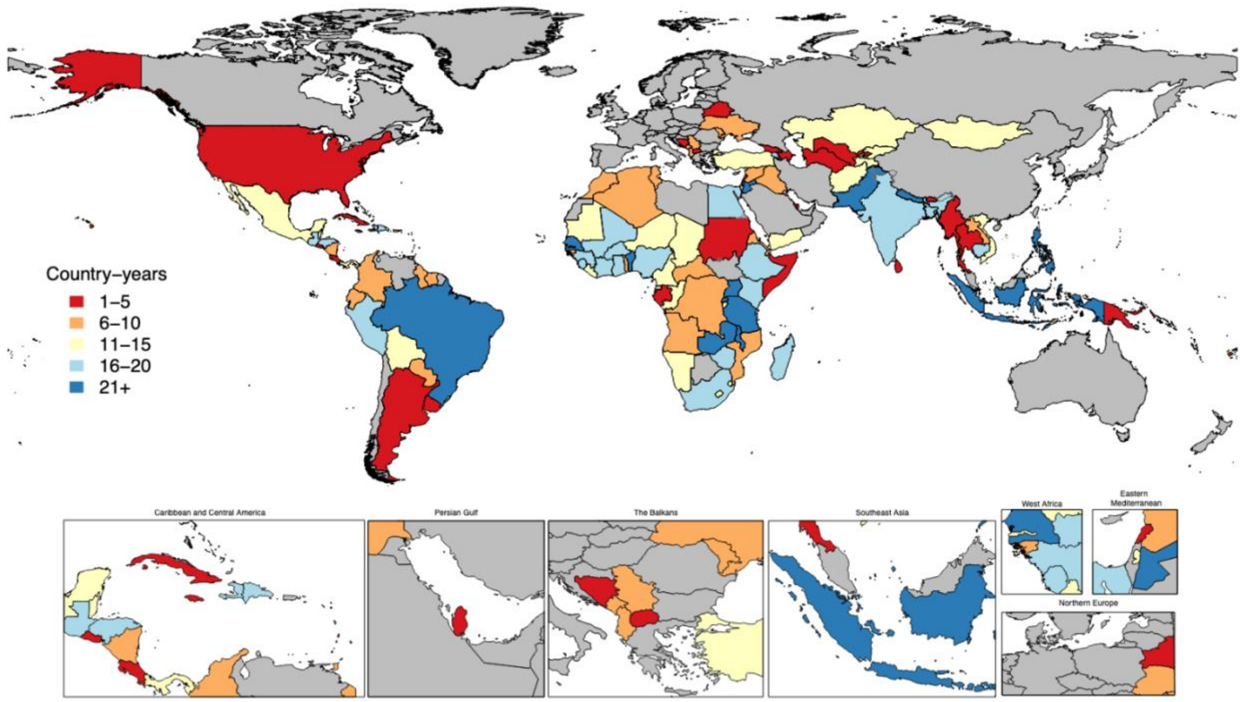

Supplementary Figure 2. Map of input data of delivery in a public hospital

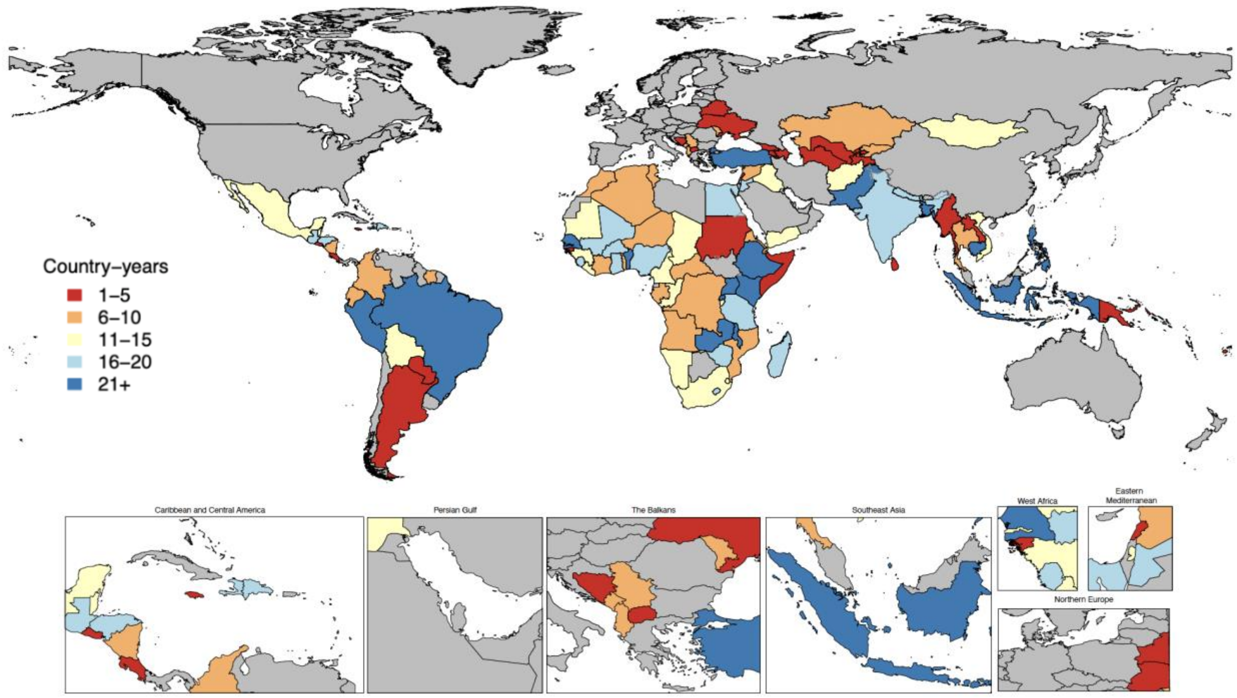

Supplementary Figure 3. Map of input data of delivery in a private hospital

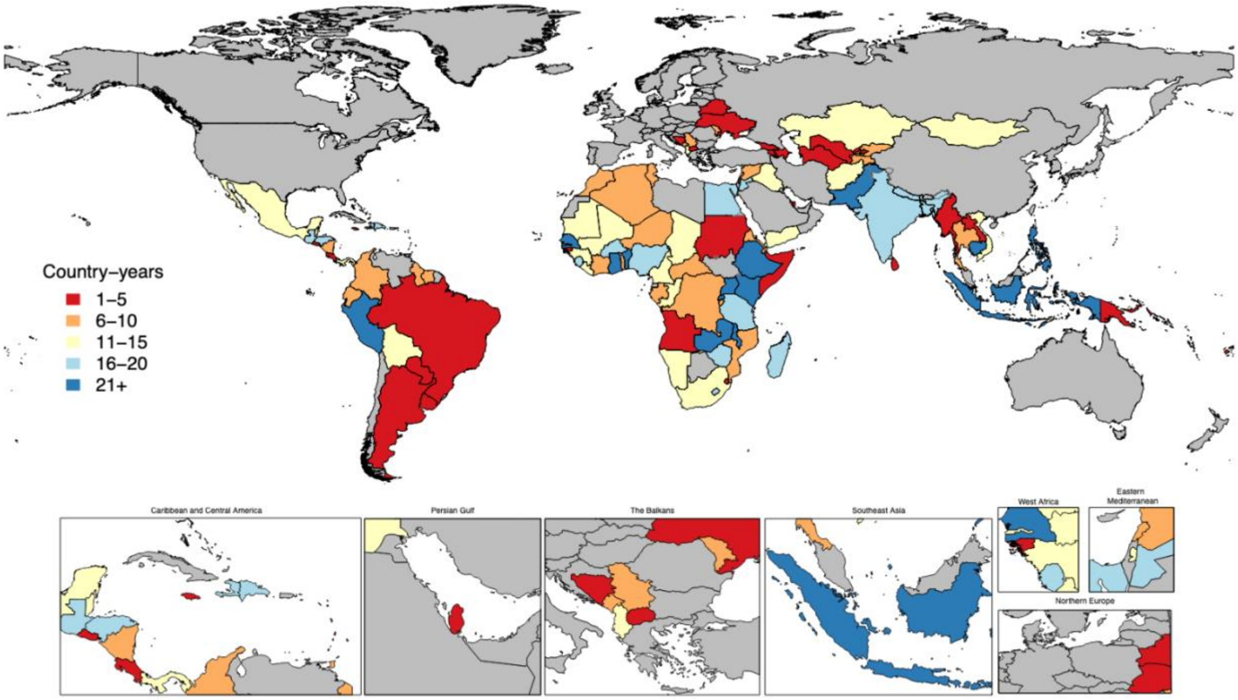

Supplementary Figure 4. Map of input data of delivery in a lower-level facility of any sector

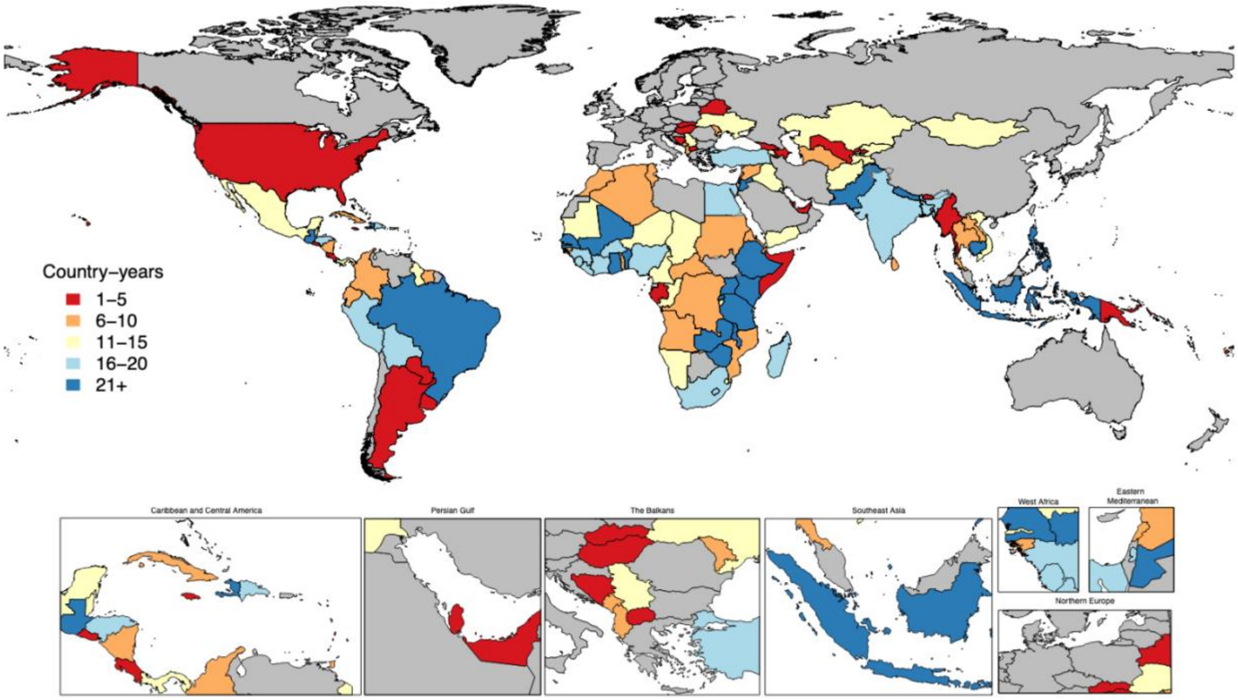

Supplementary Figure 5. Map of input data of delivery in a public lower-level facility

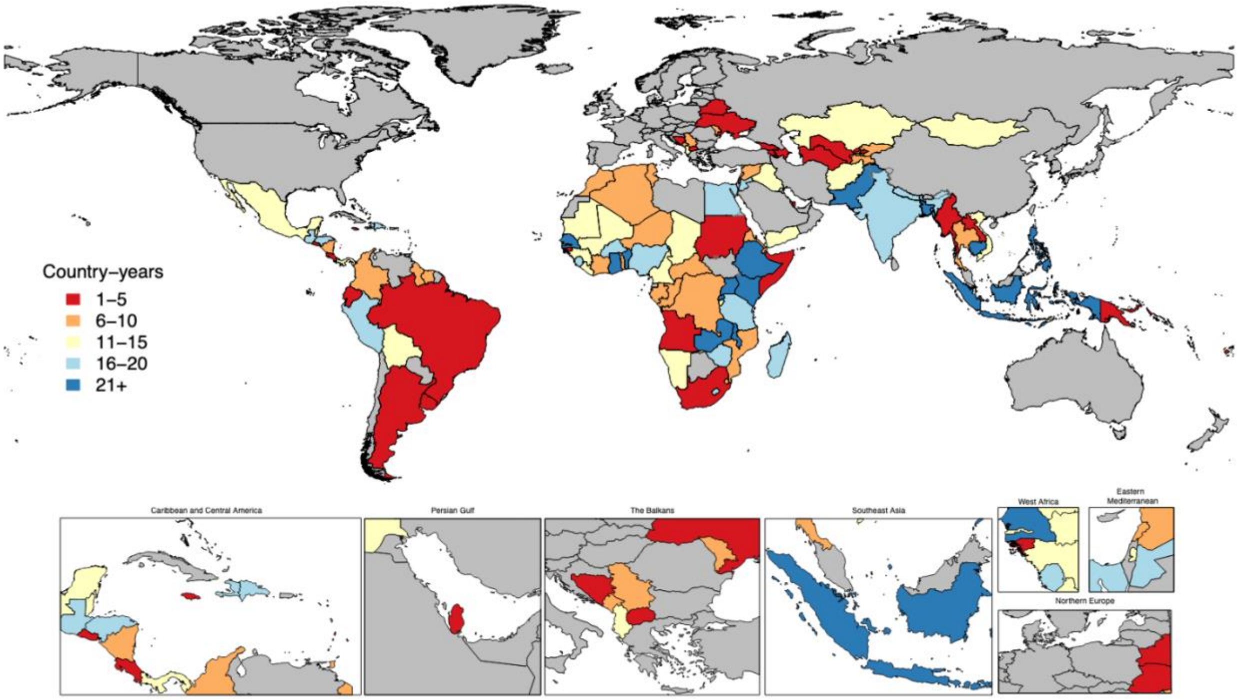

Supplementary Figure 6. Map of input data of delivery in a private lower-level facility

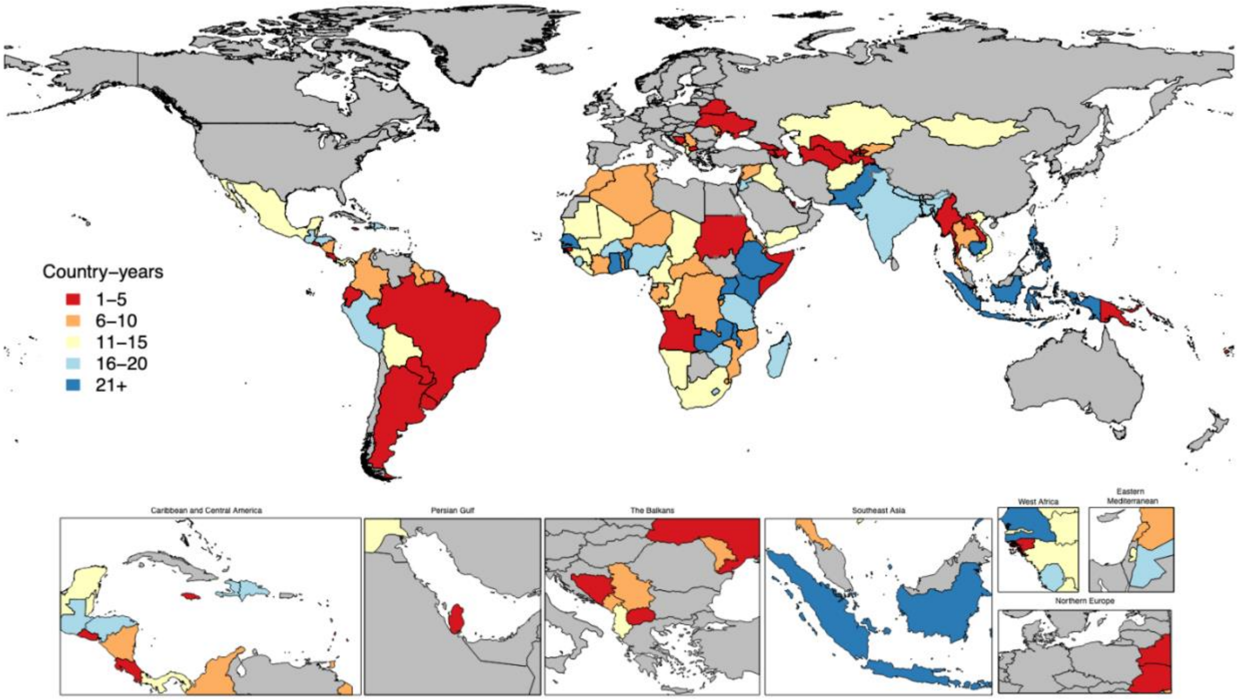

Supplementary Table 2. Data inputs by time period

N location-years	Public or non-profit hospital	Private for-profit hospital	Public or non-profit lower-level	Private for-profit lower-level	Any hospital	Any lower -level

1995-2004	443	430	431	420	477	540
2005-2014	608	562	583	555	610	653
2015-2023	222	220	211	219	225	250

Health facility categorization

We undertook a multi-step process to sort survey response options of delivery location into our categories of health facility level (hospital, lower-level or unknown) and sector (public, private for profit or private non-profit). First, we reviewed the name of the response and assigned the category based on common facility names provided in Supplementary Table 3. For example, if a woman selected the response option "Public hospital", it would be categorized into the hospital level and public sector. In the DHS and MICs surveys, response options were often grouped by sector. See Supplementary Figure 7 as an example of how we categorized these responses into sectors. In this example from the 2015 Zimbabwe Demographic and Health Survey,[2] "Urban municipal clinic" was categorized as lower-level and public sector.

Supplementary Table 3. Examples of how response options were categorized into facility level and sector

**Level**	**Response option examples**
Hospital	Hospital, hospitalier, hôpital, surgical health center, cma (centre medical avec antenne chirurgical), referral, hosp, maternity hospital, second level, royal medical services, health complex
Lower level	Dispensary, clinic, physician office, health post, health hut, health cabin, health cabinet, health house, health unit, outpatient, mobile outreach, clin, off, basic health center, primary health care center, centre de sante de base, cabinet de soins, case de sante, post, phu, clinic/health center, first level, community health center, village unit, station, cs, puskesmas/polindes/pustu, sub center, pharmacy
Level unknown based on name; conduct further investigation	Hospital/clinic; hospital/center; hospital/dispensary; nurse, doctor or midwife without specifying facility; facility in another country; health center; maternity home; maternity center; maternity clinic; nursing/maternity; polyclinic
**Sector**	**Response option examples**
Public	Public, government, govt, gouv, gouvernemental, publica, parastatal, state, district, regional, provincial, min. of health, moh, imss, military
Private for profit	Private, private for profit, prive, privee, privado, privada, pvt, prvt
Private not for profit	Non-government, ngo, Private non-profit, faith-based, mission, church, mosque, ong, foundation, fundacion, trust, religious, voluntary, marie stopes, red cross

Sector unknown based on name; conduct further investigation	Not sure, not sure if public or private, other, mixed

Supplementary Figure 7. Example of response options from the 2015 Zimbabwe Demographic and Health Survey[2] and our categorization into sector

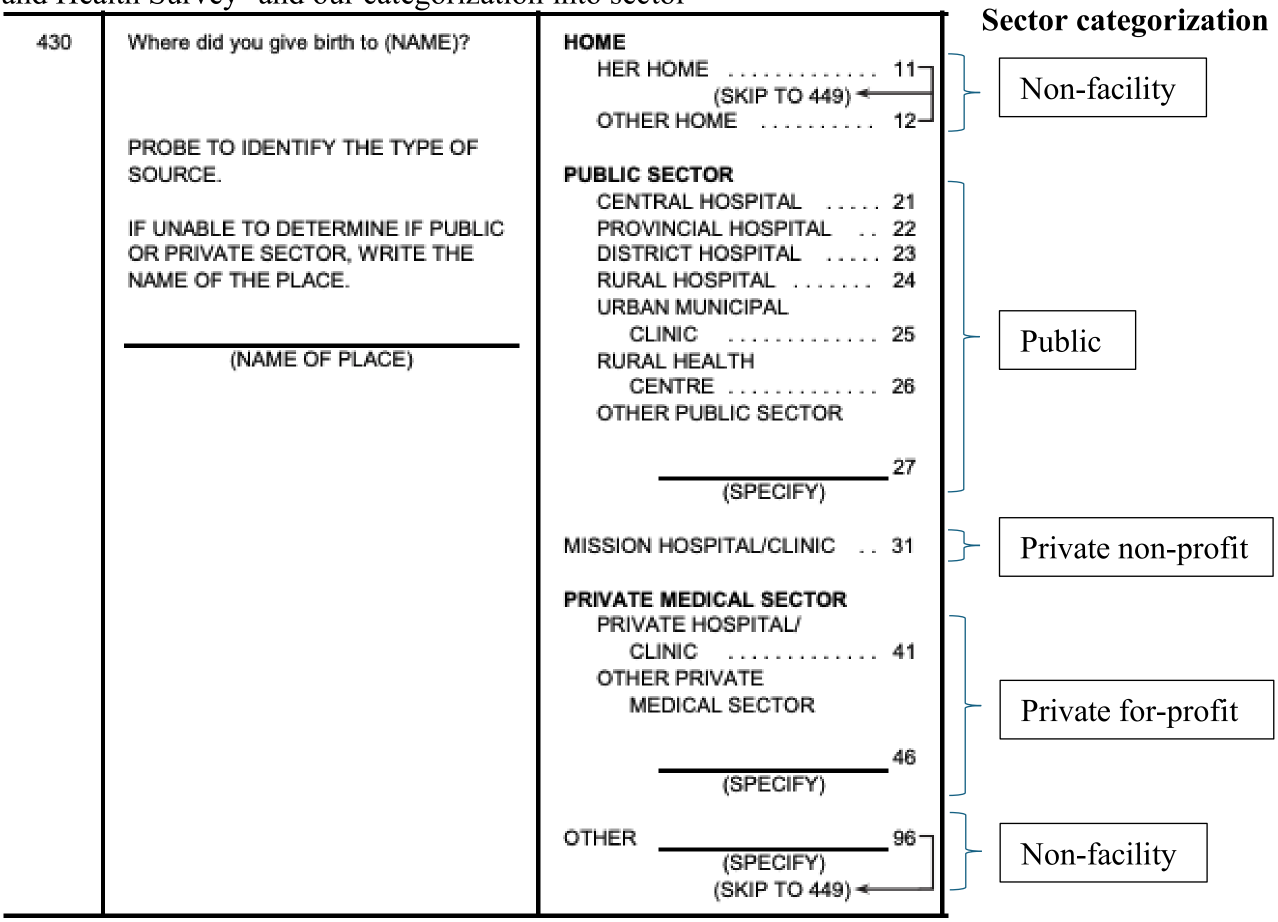

In the second step, we reviewed country-specific literature and reports to assist in categorizing a response option that was in the 'level unknown' or 'sector unknown' category. Examples of documents reviewed include the Health Systems in Transition Series, Service Availability and Readiness Assessments, or Ministry of Health documents such as reports from Health Management Information Systems.

Third, if the level was still unknown after the first two steps, we used cesarean section (c-section) rates as a sorting factor, an approach used previously for facility categorization.[3] We calculated the proportion of deliveries conducted via c-section for each response option in the survey.

Facility types with high c-section rates were assigned as hospitals as facility types with more c-sections are more likely to have operating rooms, skilled health personnel, and other characteristics typical of hospitals. For most countries, facility types with c-section rates over 12% were categorized as hospital level, facility types with under 5% were categorized as lower level and between 5% and 12% were still considered unknown. In countries where the prevailing c-section rate among all births was under 10%, we lowered the thresholds and considered more facility types to be unknown.

Fourth, we compared the categorization of all responses across surveys from the same location and adjusted the categorizations to ensure consistency across surveys. Finally, we consulted with experts on health systems across all regions to review the data sources, the categorization of facility types, and final estimates of delivery location over time. These final steps resulted in over-writing some of our previous categorizations from the first three steps, as well as further categorizing the unknown categorizations to minimize the response options that were unable to be categorized.

After categorization, there were no deliveries in private non-for-profit facilities in three-quarters of location-years and they accounted for less than 2% of deliveries in 90% of location-years. This is likely due to data limitations; in many surveys, non-profit, mission or faith-based providers were not given as response options. We assumed that private non-profit facilities in most locations are more similar in cost and quality to the public sector than the private for-profit sector.[4] We therefore combined these deliveries with the public sector for modelling.

As a sensitivity analysis, we modeled private non-profit deliveries separately, with the results for 2023 shown in Supplementary Figures 17 and 18. However, it should be cautioned that because many surveys did not give response options for non-profit facilities, these estimates may underestimate the true share of non-profit deliveries. Locations with private non-profit hospital deliveries exceeding 2% of the in-facility delivery share in 2023 were: Eswatini, Lesotho, Zimbabwe, Malawi, Cameroon, Palestine, Kenya, Papua New Guinea, and United Republic of Tanzania. Locations with private non-profit lower-level deliveries exceeding 2% of the facility delivery share in 2023 were: Papua New Guinea, Cameroon, Zambia, Malawi, and Lesotho. To group private non-profit facilities with private for-profit facilities instead of public facilities, the delivery share in these countries would be correspondingly lower for the public and non-profit category and higher in the private for-profit category.

After categorising the responses, an average of 2% of women delivered in a facility with an unknown level and 7% delivered in facility with an unknown sector. Given that we had more information about level than sector, we ran two additional envelope models for hospital deliveries and lower-level deliveries regardless of sector so that we were still able to utilize data where level was known but sector was unknown.

Modelling strategy

Supplementary Figure 8. Modeling strategy flowchart

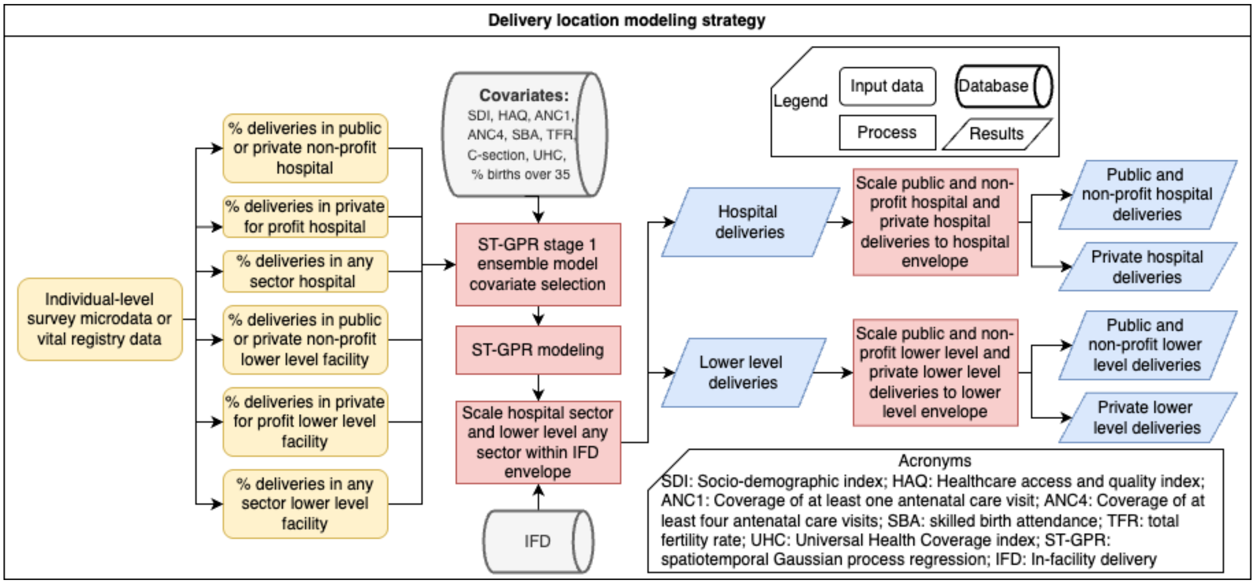

Each delivery location indicator was modelled separately using spatio-temporal process Guassian regression (ST-GPR), a modeling technique used for creating estimates across all years and geographies used frequently in the Global Burden of Disease, Risk Factors and Injuries (GBD) study.[5] ST-GPR is a three-stage process that leverages relationships between data and predictive covariates and borrows strength across geography. ST-GPR was chosen because it allows for a flexible time trend, making use of the high data density that we have for delivery location. It does not assume a definitive functional form like classical linear models, but rather follows a Gaussian process. The predicted delivery share in a country *c* at time *t* is given by:

$$p_{c,t} = g_c(t) + \epsilon_{c,t}$$

$$\text{where } g_c(t) \sim GP(m_c(t),\ Cov(g_c(t))) \text{ and } \epsilon_{c,t} \sim Normal\left(0, \sigma_p^2\right).$$

The derivation of the mean function $m_c(t)$, covariance function, $Cov(g_c(t))$ and error variance, σ_p^2 are described at length in Supplementary appendix 1 of the GBD 2021 Risk Factor study (pp. 32-36)[5] and summarized below.

In the first stage of ST-GPR, we used an ensemble approach for a linear mixed-effects model to make our results robust to the choice of covariates included in the model. We first identified a list of potential covariates (Supplementary Table 4) thought to be associated with delivery level and sector to use in a linear mixed-effects model. For each delivery location indicator, we tested every combination of the covariates in mixed-effects linear regressions with nested random effects at the super-region, region and country levels according to the GBD location hierarchy.[6] We did not consider interactions between the covariates. We specified an expected direction for the coefficients and eliminated models with coefficients in the incorrect direction. We then ranked each of these models by their out-of-sample root-mean-square-error (RMSE). We selected the top fifty ranked models and created a weighted average of the models where the estimated coefficients were statistically significant. Using these ensemble models, we predicted delivery location for all location-years. Models were conducted in logit space, delivery location

proportions of exactly 0 or exactly 1 were offset by 0.00001 to enable modeling. The distribution of the coefficients for each outcome in the ensemble models are included in Supplementary Figures 9-14.

In the second stage of ST-GPR, we smoothed residuals between the prediction and the input data across location and time, producing an updated time series for each location. We smooth by assigning time and space weights to data based on how close it is in time or space to missing demographics. Weights are then multiplied by the residuals and added back into the linear prediction. This stage enables us to borrow information from data rich locations and time periods to inform areas of data sparsity. The mean function $m_c(t)$ is expressed as $logit(p_c(t) = X_c\beta + h(r_{c,t})$, where $X\beta$ is the summation of the components of the hierarchical mixed-effects regression from the first stage and $h(r_{c,t})$ is the smoothing function for the residuals.

In the third stage of ST-GPR, Gaussian process regression improves predictions for countries and years that have input data available and incorporates uncertainty in the form of draws.
The covariance function $Cov(g_c(t))$ uses the Matern-Euclidian covariance function, incorporating information on the deviation of the difference of the first-stage linear regression estimates and the second-stage spatiotemporal smoothing step. We sampled 1000 draws from the Gaussian process regression.

Finally, the error variance σ_p^2 is the sampling error variance transformed into logit-space using the delta approximation $\sigma_p^2 \cong \frac{\sigma_p^2}{\left(p_{c,t}*(1-p_{c,t})\right)^2}$.

After ST-GPR modelling, we conducted extensive visual inspection of the results. We identified input data sources that could be considered outliers when they differed substantially from other data inputs in the same country or region. We reviewed the response option categorization again for all outliers and updated it as necessary, or if it could not be improved to be more consistent with the rest of the series, removed the data point from the modelling process.

We used all possible input data and modeled estimates for all locations in the GBD hierarchy. However, due to a lack of data in high income countries and China, we reported the estimates for 130 locations, corresponding to the World Bank designated low- and middle-countries from 2025.[7]

Supplementary Table 4. Covariates considered in ensemble models

Indicator	Definition
Socio-demographic Index	A measure of development estimated via principal component analysis using log-transformed lag-distributed income, total fertility rate (<25), and education years per capita over age 15[8]
Healthcare access and quality Index	An index of scaled mortality-to-incidence ratios and risk-standardised death rates for 32 causes of death that should not occur in the presence of timely, quality health care[9]
ANC1	Proportion of pregnant women receiving any antenatal care from a skilled provider[10]

ANC4	Proportion of pregnant women receiving 4 or more antenatal care visits including 1 or more from a skilled provider[10]
Skilled birth attendance	Percent of women giving birth with a skilled birth attendant (mainly nurses, doctors, midwives)[10]
Total fertility rate	Total Fertility Rate[11]
C-section rate	Proportion of live births delivered by Caesarean Section (c-section)[10]
Universal health coverage Index	An index of 23 effective coverage measures spanning promotion, preventative and curative care[12]
Percent of births over 35	Proportion of live births by mothers age 35 and older[11]

Supplementary Figure 9. Distribution of ensemble model coefficients for hospital any sector

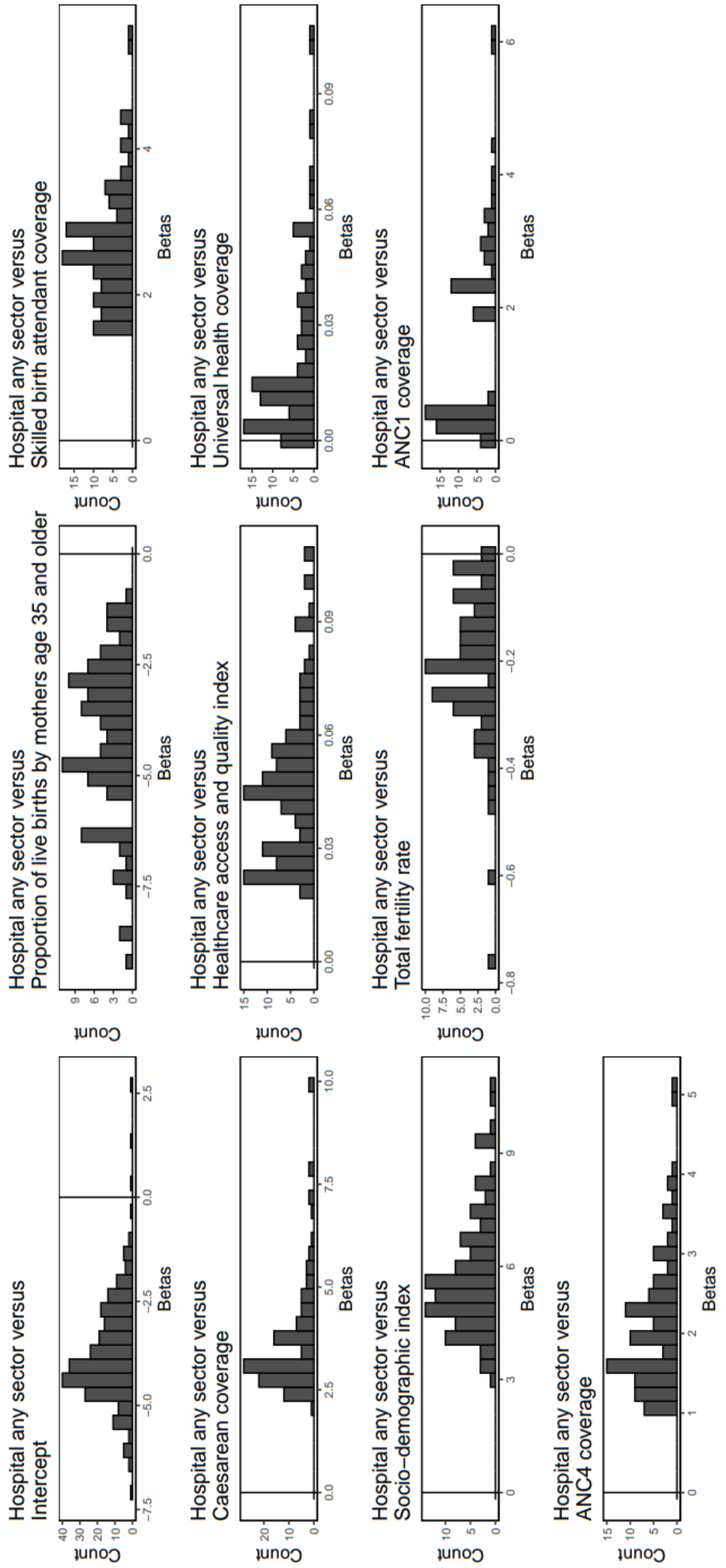

Supplementary Figure 10. Distribution of ensemble model coefficients for lower level any sector

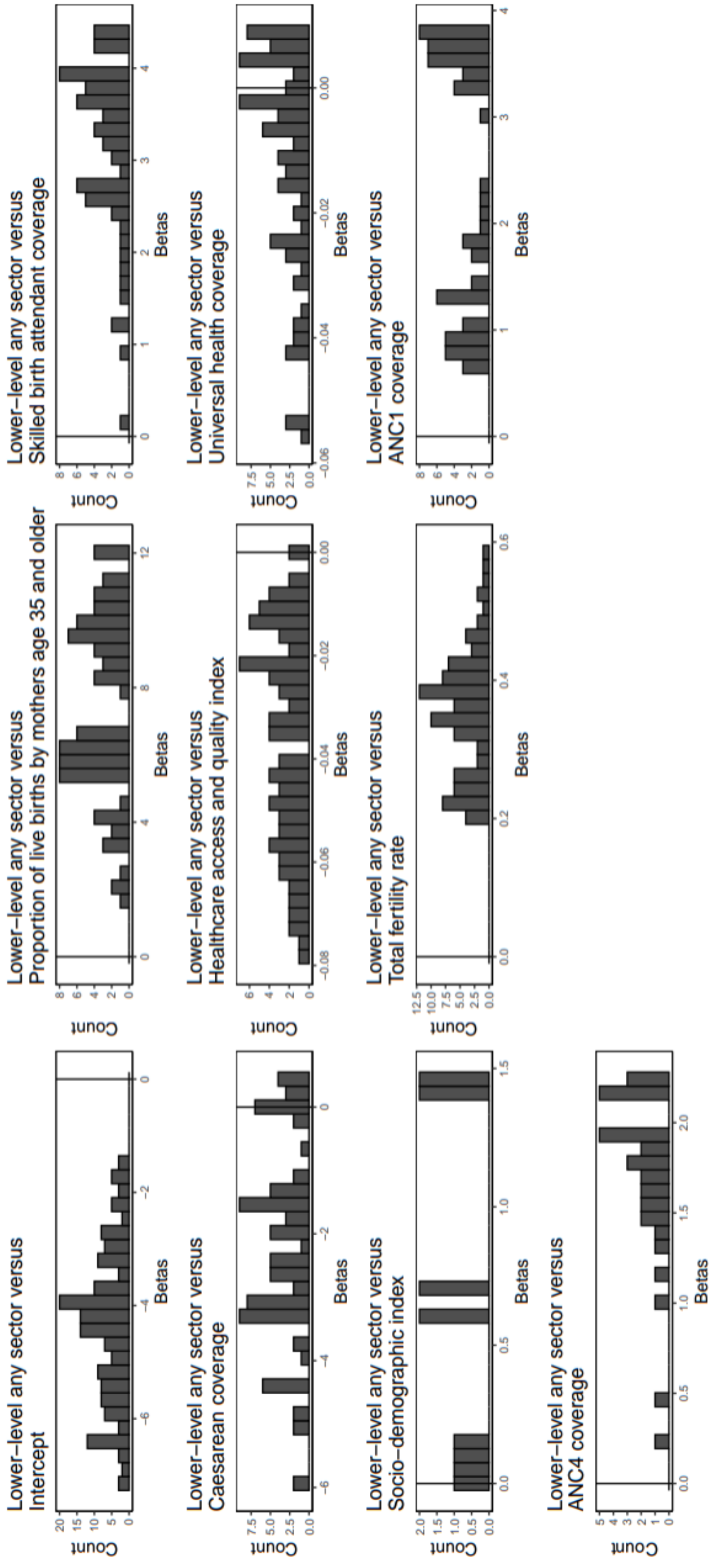

Supplementary Figure 11. Distribution of ensemble model coefficients for public hospital

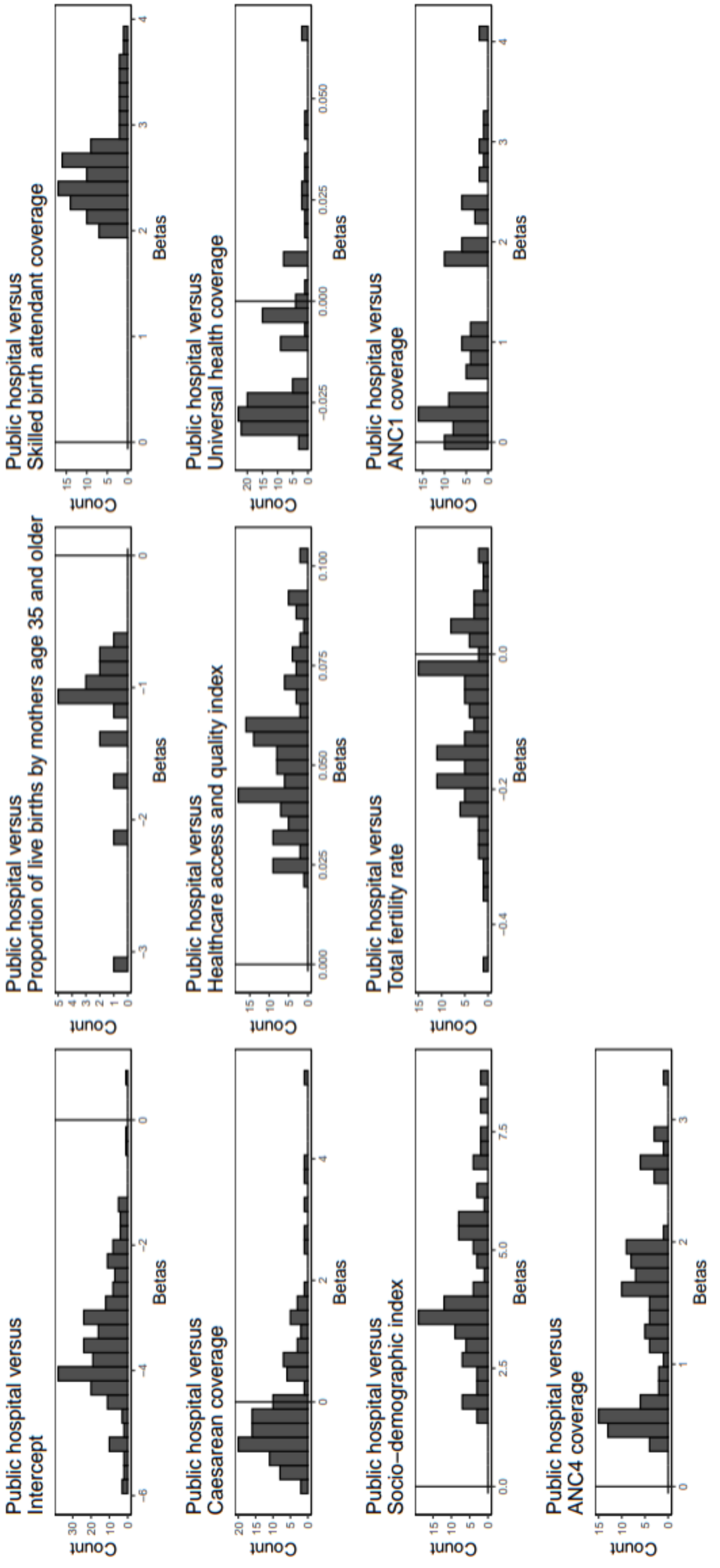

Supplementary Figure 12. Distribution of ensemble model coefficients for public lower level

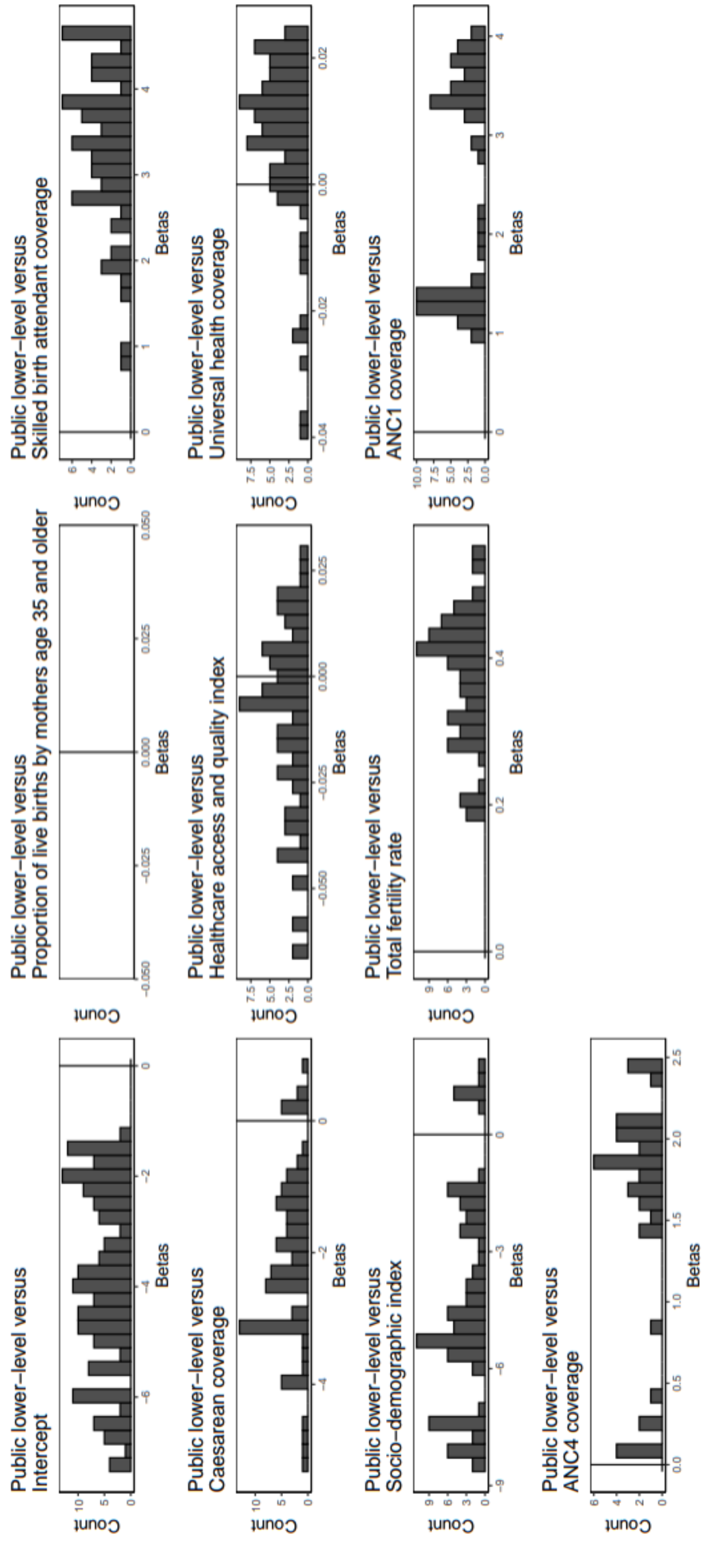

Supplementary Figure 13. Distribution of ensemble model coefficients for private hospital

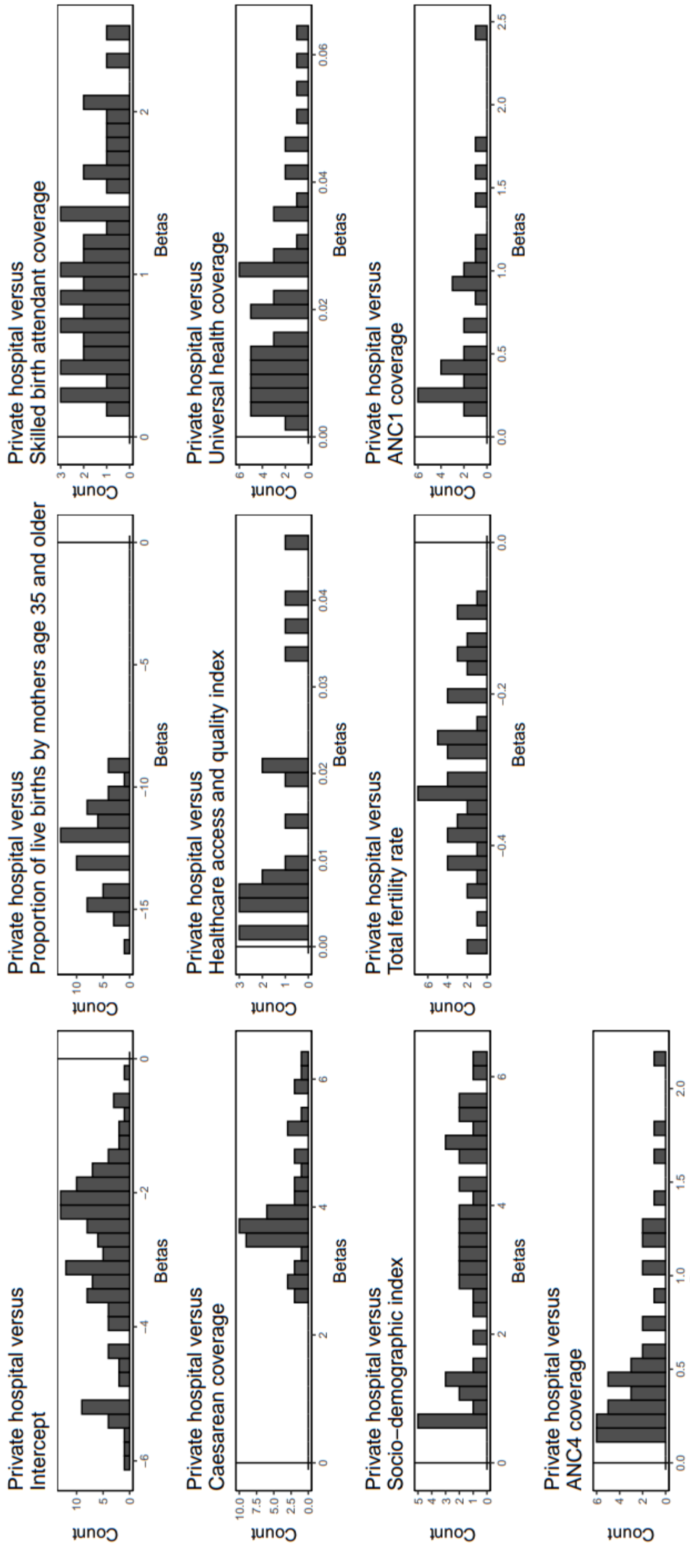

Supplementary Figure 14. Distribution of ensemble model coefficients for private lower level

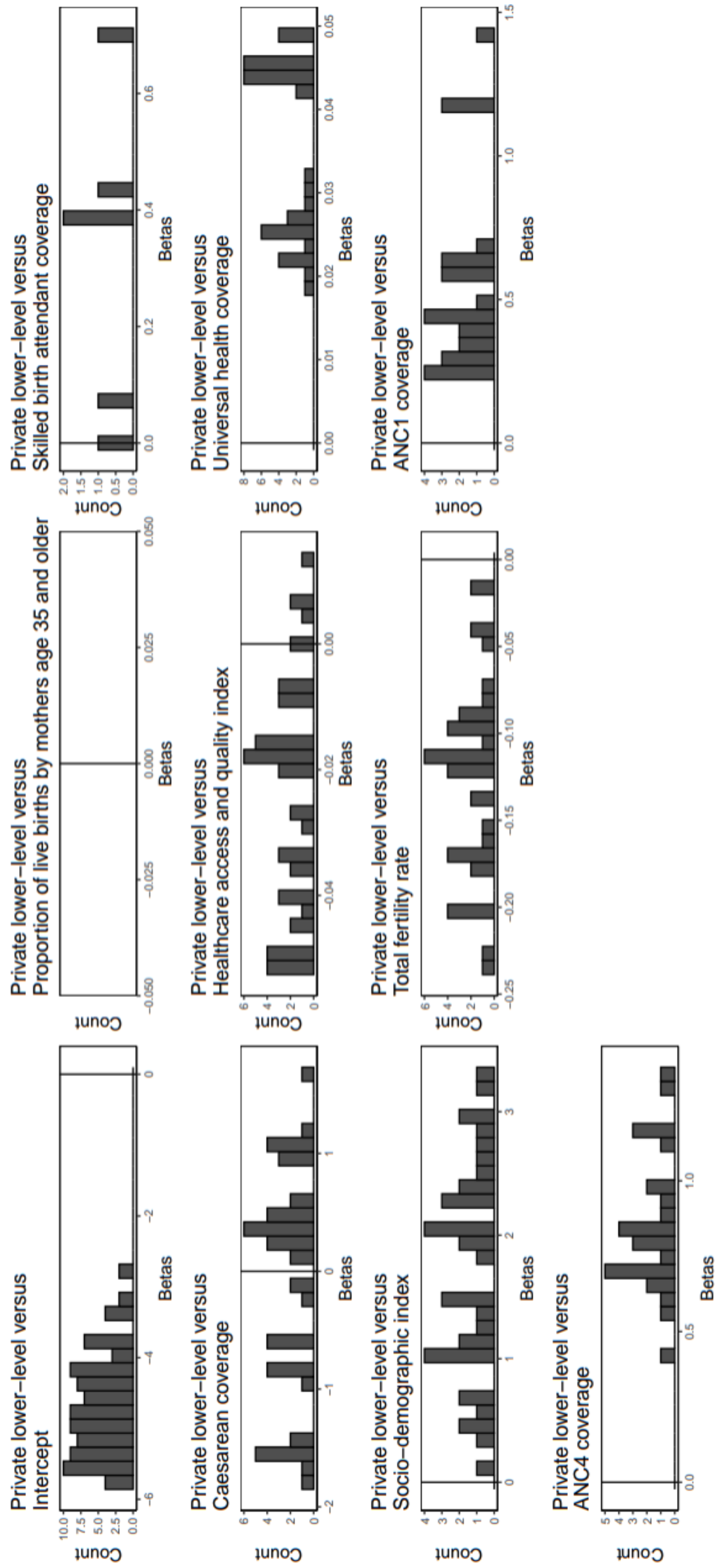

After modeling each delivery location separately, we first summed the two level-specific outcomes (any hospital and any lower-level) and scaled the results to fit within the GBD total in-facility delivery envelope.[10] We then summed the level and sector-specific outcomes to fit within the hospital and lower-level envelopes respectively. Supplementary figure 15 shows the difference between the pre- and post-scaled estimates, with negative values indicating that the pre-scaled values exceeded the envelopes while positive values indicate pre-scaled values were less than the envelope.

Supplementary Figure 15. Absolute difference between pre- and post-scaled estimates, 2023

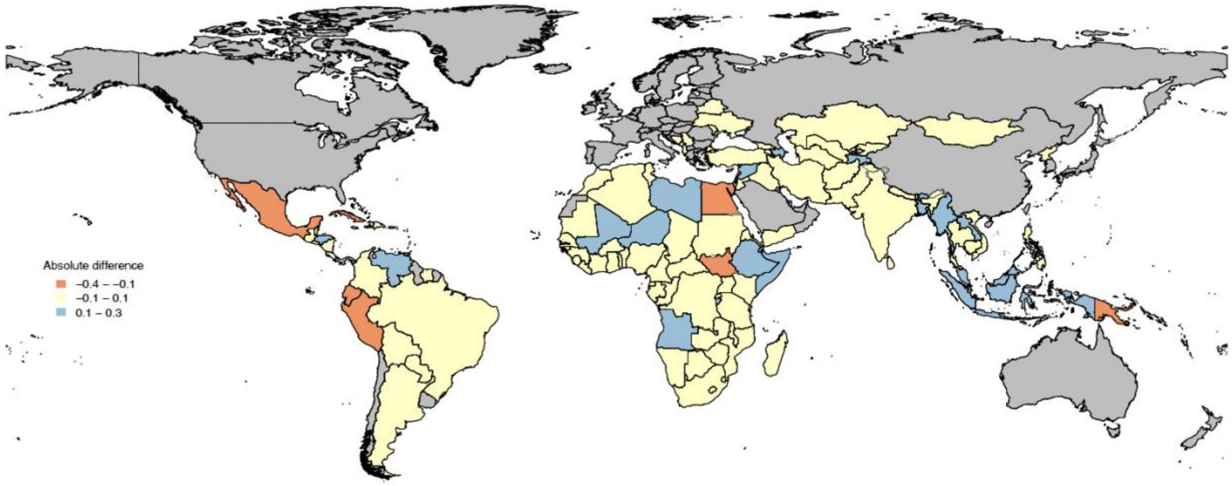

Analysis

We conducted an analysis of delivery location by a country's socio-demographic index and maternal mortality level using the Bayesian meta-regression tool MR-BRT (Meta-Regression with Bayesian priors, Regularization and Trimming).[13] The socio-demographic index and maternal mortality estimates were both accessed from the GBD 2023 study.[14] An additional analysis of the neonatal mortality rate is included in Supplementary Figure 17. The data used for the analysis was the complete estimates of delivery location for all locations in 2023. Before modeling, delivery location was transformed into logit space; this transformation removed the constraint between zero and one. We ran a separate MR-BRT model for each outcome and delivery location, though each model followed the same format: an intercept and a quadratic spline on the delivery location indicator with location random effects. We used 7 internal knots, with the knots placed at equal values of data frequency across the observed delivery location values. We then scaled the results so that they summed to 100% of all births.

Part 2. Supplementary results

Private for-profit lower-level deliveries, 2023

Supplementary Figure 16. Private for-profit lower-level deliveries as a share of facility deliveries, 2023

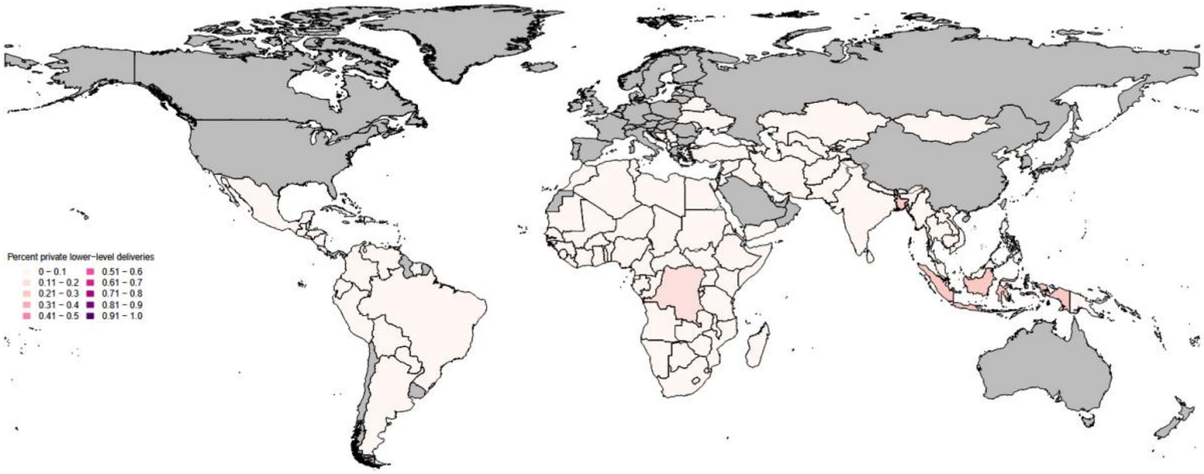

Private non-profit deliveries, 2023

Supplementary Figure 17. Private non-profit hospital deliveries as a share of facility deliveries, 2023

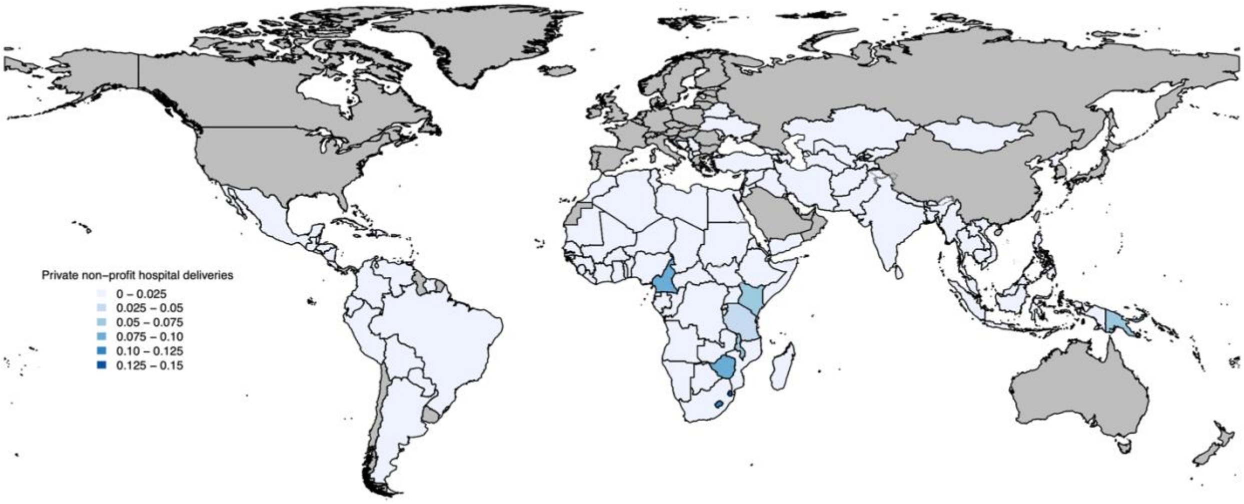

Supplementary Figure 18. Private non-profit lower-level deliveries as a share of facility deliveries, 2023

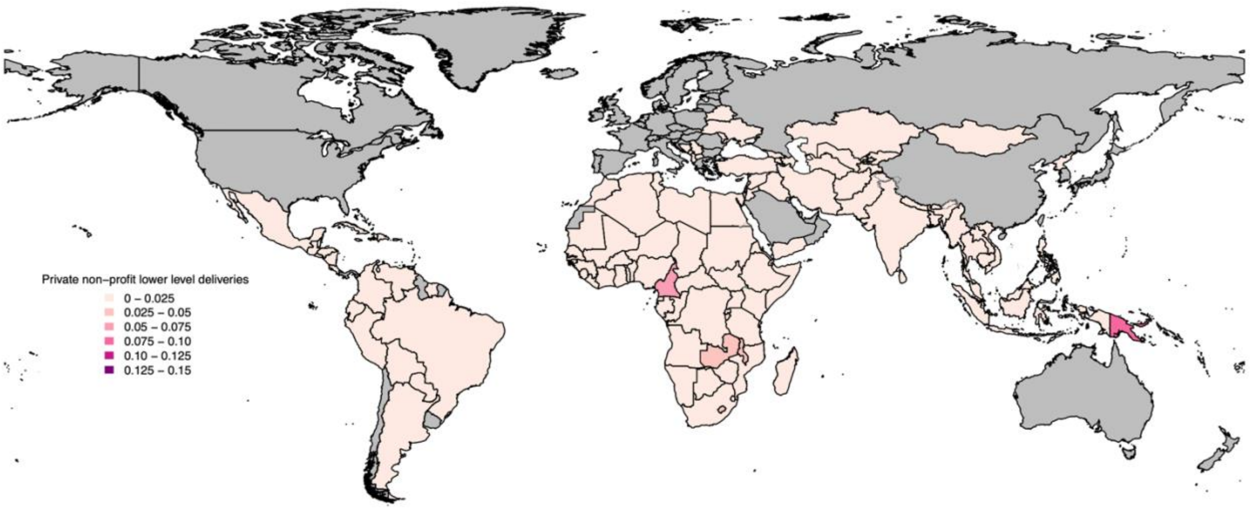

Numbers of deliveries by location, 2023

Supplementary Table 5. Number of births in thousands by location, 2023

Location	Public and private non-profit hospital	Private for-profit hospital	Public and private non-profit lower-level	Private for-profit lower-level	Non-facility
**All study countries**	**49492 (48299–50653)**	**20029 (19056–21042)**	**13547 (12793–14404)**	**2121 (1929–2329)**	**18951 (18012–19998)**
**Central Europe, Eastern Europe, and Central Asia**	**2436 (2419–2450)**	**17·7 (12–25·5)**	**51·5 (43·2–61·1)**	**4·6 (2·8–7·5)**	**27·5 (20–39·3)**
Albania	23·4 (23·2–23·5)	0·2 (0·1–0·2)	0 (0–0)	0 (0–0)	0·2 (0·1–0·4)
Armenia	14·3 (12·5–16·7)	0·7 (0·4–1·1)	19·3 (16·9–21·2)	0 (0–0)	0 (0–0)
Azerbaijan	117 (112–121)	1·5 (0·8–2·3)	3·3 (1·5–5·9)	2 (0·9–3·9)	3·7 (1·7–7·1)
Belarus	76·8 (76·7–76·8)	0 (0–0)	0 (0–0·1)	0·1 (0–0·1)	0 (0–0)
Bosnia and Herzegovina	22·9 (21·4–24)	0 (0–0)	2·2 (1·1–3·6)	0 (0–0·1)	0·1 (0–0·1)
Georgia	38·5 (37·4–39·5)	1·7 (1–2·7)	0·2 (0·1–0·5)	0·9 (0·4–1·7)	0·1 (0·1–0·2)
Kazakhstan	399 (398–399)	0·1 (0–0·1)	0·2 (0·1–0·4)	0·2 (0·1–0·4)	0·2 (0·1–0·3)
Kyrgyzstan	147 (147–148)	1 (0·6–1·5)	0·2 (0·1–0·3)	0·4 (0·2–0·7)	0·4 (0·2–0·6)
Mongolia	65·7 (61·1–69·4)	0·4 (0·2–0·5)	8·3 (4·6–12·9)	0·1 (0–0·2)	0·2 (0·1–0·4)
Montenegro	6·9 (6·9–6·9)	0 (0–0)	0 (0–0·1)	0 (0–0)	0 (0–0)
North Macedonia	13·9 (12·6–15·1)	0·3 (0·2–0·5)	2·8 (1·7–4·2)	0 (0–0·1)	0 (0–0)

Republic of Moldova	25·1 (24·9–25·2)	0 (0–0)	0·1 (0–0·2)	0·1 (0–0·2)	0·1 (0–0·2)
Serbia	60 (59·4–60·3)	0·1 (0·1–0·2)	0·7 (0·3–1·2)	0·1 (0–0·1)	0 (0–0)
Tajikistan	256 (244–265)	2·1 (1·2–3·4)	2·6 (1·2–4·8)	0·1 (0–0·1)	20 (11·1–32·1)
Turkmenistan	111 (107–114)	0·1 (0·1–0·2)	10 (6·8–14·3)	0·1 (0–0·1)	0 (0–0)
Ukraine	213 (213–213)	0·4 (0·3–0·7)	0·4 (0·2–0·8)	0 (0–0·1)	0 (0–0)
Uzbekistan	845 (840–850)	9 (5·3–14·7)	1·1 (0·5–2·2)	0·4 (0·2–0·9)	2·5 (1·3–4·3)
**Latin America and Caribbean**	**5611 (5470–5747)**	**1808 (1696–1929)**	**530 (455–606)**	**173 (146–209)**	**503 (420–602)**
Argentina	322 (301–344)	159 (138–181)	12·9 (8·2–19·8)	0·6 (0·3–1·1)	1·2 (0·6–2·1)
Belize	5·7 (5·4–6·1)	0·6 (0·4–0·9)	0 (0–0)	0·3 (0·2–0·6)	0·3 (0·1–0·5)
Bolivia (Plurinational State of)	152 (131–172)	33·6 (21·6–47·7)	19·2 (10·5–31·5)	2·2 (0·9–4·5)	26·1 (13·2–46·2)
Brazil	2153 (2051–2258)	798 (692–898)	23·6 (20·3–27)	0·3 (0·2–0·5)	12·8 (5·9–24·1)
Colombia	504 (456–551)	181 (132–227)	19·4 (9·2–36·1)	1 (0·4–2·2)	10·1 (5·2–17·9)
Costa Rica	49·6 (49·1–50·1)	1·1 (0·7–1·6)	0·3 (0·2–0·5)	0·1 (0–0·2)	0·1 (0–0·2)
Cuba	73·9 (67·9–79·6)	18·7 (12·9–24·7)	0 (0–0)	0 (0–0·1)	0·1 (0–0·1)
Dominica	0·5 (0·5–0·6)	0·1 (0·1–0·1)	0 (0–0)	0 (0–0)	0 (0–0)
Dominican Republic	165 (151–177)	39·1 (26·7–53·2)	0·1 (0–0·2)	0·2 (0·1–0·4)	2·1 (1·2–3·5)
Ecuador	182 (159–204)	103 (80·2–123)	4·2 (1·8–8·1)	9·7 (4·6–17·7)	2·4 (1·8–3)
El Salvador	77·8 (75·9–79·3)	2·3 (1·5–3·4)	0·4 (0·2–0·6)	0·1 (0·1–0·3)	2·1 (1–3·8)
Grenada	1·2 (1·1–1·3)	0·2 (0·1–0·2)	0 (0–0)	0 (0–0)	0 (0–0)
Guatemala	190 (176–203)	29·3 (19·6–40·7)	29 (23·8–34·4)	4 (2·2–6·5)	85·8 (77·2–95)
Haiti	105 (67·5–147)	14·3 (7·5–23·3)	32·8 (18–52)	3 (1·3–5·7)	200 (148–250)
Honduras	195 (183–205)	3·4 (2·3–4·8)	7·9 (4·1–13·2)	16·6 (10·2–25·1)	16·6 (10·3–25·1)
Jamaica	33·1 (31·7–34·3)	2·7 (1·6–4)	0 (0–0·1)	0·1 (0–0·1)	0·8 (0·4–1·5)
Mexico	912 (844–977)	404 (370–439)	292 (221–368)	73·7 (48·6–107)	113 (87·5–148)
Nicaragua	105 (95·7–112)	1·2 (0·7–2)	1·1 (0·4–2·3)	5·2 (2·5–9·3)	11·4 (5·6–19·6)
Paraguay	59·6 (50·5–68·4)	30·4 (22·9–38·3)	12·2 (6·9–18·5)	1·4 (0·6–2·8)	4·2 (2·1–7·5)
Peru	364 (323–404)	58 (43·8–75·1)	91·5 (63·1–124)	60·6 (39·9–86·3)	33·4 (21·7–49·3)

Saint Lucia	1·7 (1·7–1·7)	0 (0–0·1)	0 (0–0)	0 (0–0)	0 (0–0)
Saint Vincent and the Grenadines	1·1 (1·1–1·2)	0·1 (0·1–0·2)	0 (0–0)	0 (0–0)	0 (0–0)
Suriname	5·5 (5–6·1)	2·1 (1·7–2·6)	0·8 (0·5–1·2)	0·1 (0–0·2)	0·5 (0·3–0·8)
Venezuela (Bolivarian Republic of)	274 (255–290)	30·4 (18·8–45·1)	13 (6·2–23·6)	3·5 (1·3–7)	8·5 (3·8–16·1)
**North Africa and Middle East**	**5750 (5497–5999)**	**2349 (2190–2504)**	**654 (590–724)**	**92·9 (80·5–107)**	**1705 (1462–1950)**
Algeria	717 (677–754)	55·2 (38·2–77·4)	91·5 (59·8–130)	1·4 (0·7–2·6)	7·3 (3·9–12·7)
Egypt	904 (795–1036)	1077 (949–1185)	14·6 (7·1–27·3)	10·7 (4·7–21·3)	94·1 (47·8–161)
Iran (Islamic Republic of)	727 (659–794)	224 (157–293)	2·7 (1·1–5·7)	4·5 (1·9–8·6)	8·3 (3·8–15·9)
Iraq	711 (662–755)	133 (99·6–173)	0·9 (0·4–1·6)	1·2 (0·6–2)	68·7 (39·7–108)
Jordan	143 (131–156)	77·4 (64–89·4)	0·1 (0–0·2)	0·1 (0·1–0·2)	2·8 (2·1–3·6)
Lebanon	16·5 (14·4–18·7)	64 (61·9–66·1)	0 (0–0·1)	1 (0·5–1·8)	0·2 (0·1–0·4)
Libya	99·7 (94·2–104)	11·1 (6·8–16·5)	1·3 (0·5–2·6)	1·5 (0·6–2·8)	0·2 (0·1–0·4)
Morocco	370 (328–408)	22·7 (13·5–35·5)	80·8 (44·4–121)	1 (0·4–2)	26·8 (12·4–49·7)
Palestine	86·8 (80·4–93·1)	40 (33·4–46·1)	0·2 (0·1–0·4)	2·7 (1·6–4·2)	0·2 (0·1–0·3)
Syrian Arab Republic	152 (127–175)	48·4 (31·2–68·2)	1 (0·4–2·1)	14·6 (7·4–25)	33·9 (16·2–57·2)
Tunisia	123 (116–130)	26·3 (19·4–34·1)	0·1 (0·1–0·2)	0·1 (0·1–0·2)	0·7 (0·4–1·1)
Türkiye	545 (486–608)	348 (291–404)	62·7 (36·6–98)	6·9 (3·3–12·8)	19·7 (9·2–35·9)
Yemen	311 (281–341)	98·7 (84·8–115)	91·4 (75·8–109)	15·7 (11·9–20·2)	535 (490–578)
**South Asia**	**16842 (16432–17253)**	**8722 (8343–9128)**	**1896 (1801–2040)**	**386 (309–463)**	**3212 (3119–3353)**
Afghanistan	490 (457–523)	106 (92·5–121)	304 (278–332)	30·6 (25·6–36·7)	416 (375–459)
Bangladesh	1033 (888–1185)	513 (390–632)	210 (138–296)	587 (458–721)	935 (825–1056)
Bhutan	5·9 (5–6·9)	1·7 (1·1–2·4)	1·3 (0·8–2)	0·2 (0·1–0·3)	1·3 (0·6–2·1)
India	12387 (12082–12679)	5773 (5522–6036)	1439 (1365–1574)	30·5 (25·6–36·5)	1640 (1458–1898)
Nepal	262 (242–281)	91·8 (79·7–105)	113 (98·5–128)	1·7 (1·3–2·2)	111 (92·3–132)
Pakistan	2301 (1987–2634)	2297 (1989–2615)	14·7 (8·5–23·8)	1·3 (0·6–2·4)	1308 (915–1765)
**Southeast Asia, East Asia, and Oceania**	**5514 (5233–5831)**	**1604 (1324–1914)**	**1552 (1348–1782)**	**1250 (975–1540)**	**1471 (1272–1738)**
Cambodia	128 (116–138)	20·5 (15·3–26·6)	168 (157–179)	35·2 (29·9–41·9)	8·9 (6·1–12·3)

Democratic People's Republic of Korea	221 (201–236)	29·6 (18·2–43·1)	18·7 (9·1–32·6)	1·6 (0·6–3·3)	18·2 (11·1–28)
Fiji	17·1 (16·9–17·3)	0 (0–0)	0·7 (0·5–0·9)	0 (0–0)	0 (0–0)
Indonesia	1229 (986–1525)	833 (618–1087)	635 (437–848)	1183 (913–1470)	474 (284–726)
Kiribati	2·2 (2–2·4)	0 (0–0)	0·5 (0·4–0·7)	0 (0–0)	0·4 (0·2–0·6)
Lao People's Democratic Republic	97·8 (78·2–114)	0·3 (0·2–0·5)	19·6 (12·4–28·5)	2·8 (1·3–5·1)	49 (32·2–69·5)
Malaysia	352 (317–381)	19·6 (11·5–30·5)	49·6 (26·7–79·1)	12·5 (5·2–23·7)	2·7 (1·2–5)
Maldives	4·9 (4·6–5·3)	1·1 (0·8–1·4)	0·1 (0·1–0·2)	0 (0–0)	0·1 (0·1–0·2)
Marshall Islands	0·8 (0·7–0·8)	0 (0–0)	0 (0–0·1)	0 (0–0)	0 (0–0)
Micronesia (Federated States of)	1·6 (1·4–1·7)	0 (0–0)	0·1 (0–0·1)	0 (0–0)	0·2 (0·1–0·4)
Myanmar	602 (469–737)	95·1 (57·2–144)	38·9 (18·9–69)	2·1 (0·8–4·5)	359 (214–513)
Papua New Guinea	123 (88·6–160)	4·1 (2·2–6·7)	100 (69·2–133)	0·3 (0·2–0·5)	139 (91·1–195)
Philippines	810 (733–892)	486 (421–550)	316 (254–388)	3·2 (2·2–4·4)	365 (295–445)
Samoa	4·8 (4·5–5·1)	0 (0–0)	0·6 (0·4–0·8)	0 (0–0)	0·6 (0·4–1)
Solomon Islands	20·1 (18·2–21·6)	0 (0–0·1)	2 (1–3·4)	0·1 (0–0·1)	1·9 (0·9–3·2)
Sri Lanka	254 (252–256)	0·8 (0·5–1·4)	0 (0–0)	2·9 (1·4–5)	0·4 (0·2–0·8)
Thailand	453 (443–462)	39·2 (30·4–49·3)	0·5 (0·2–0·9)	1·7 (1–2·7)	1·7 (1–2·5)
Timor-Leste	16 (11·3–20·8)	0·3 (0·2–0·5)	8·1 (5–11·6)	0·1 (0·1–0·2)	17·4 (11·6–23·7)
Tonga	2·2 (2·1–2·2)	0 (0–0)	0 (0–0)	0 (0–0)	0 (0–0·1)
Vanuatu	7·6 (6·9–8·2)	0 (0–0)	0·6 (0·3–1·1)	0 (0–0)	0·9 (0·4–1·5)
Viet Nam	1157 (1080–1226)	72·2 (50·8–101)	192 (128–272)	4·4 (2·4–7·6)	30·7 (18·4–50·1)
**Sub-Saharan Africa**	**12862 (12135–13638)**	**4260 (3731–4808)**	**9387 (8686–10144)**	**451 (346–585)**	**12526 (11820–13356)**
Angola	514 (384–652)	14·4 (8·1–23·5)	302 (199–414)	3 (1·5–5·5)	420 (259–604)
Benin	240 (223–258)	38·1 (31–46·6)	197 (178–215)	0·6 (0·4–0·7)	36·8 (28·3–47·5)
Botswana	41·5 (38–44·1)	2 (1·2–3·1)	5·3 (2·8–8·7)	0·2 (0·1–0·4)	0·1 (0–0·2)
Burkina Faso	98·7 (87·7–111)	2·9 (1·8–4·8)	853 (830–872)	3·3 (2·7–4)	59·1 (42·7–79·4)
Burundi	179 (144–221)	7·8 (4·5–12·4)	230 (187–267)	17·6 (11·7–25·8)	72·1 (42·2–115)

Cabo Verde	4·5 (3·9–5)	0·9 (0·6–1·3)	0·9 (0·5–1·5)	0 (0–0)	0·4 (0·2–0·8)
Cameroon	427 (341–521)	64·7 (38·1–100)	333 (244–416)	1·5 (0·9–2·6)	265 (183–361)
Central African Republic	97·7 (74·9–123)	8·7 (5·4–13·2)	76·9 (55·6–98·5)	3·3 (1·9–5)	100 (69·4–135)
Chad	183 (131–248)	13·4 (7·9–21·5)	81·1 (50·1–121)	2·2 (1·2–4)	658 (569–734)
Comoros	17·1 (16·5–17·8)	0·9 (0·7–1·1)	3·7 (3·1–4·4)	0 (0–0)	1 (0·7–1·3)
Congo	97·8 (88·5–106)	5·3 (3·3–7·9)	22·3 (13·7–32·4)	15 (12·5–17·8)	7·5 (3·6–13·5)
Côte d'Ivoire	493 (445–539)	38 (27·4–50·5)	392 (345–438)	8·9 (6·7–11·5)	199 (159–247)
Democratic Republic of the Congo	1052 (800–1330)	572 (389–789)	782 (558–1024)	340 (224–492)	374 (230–560)
Djibouti	31·9 (30·3–33·2)	0·6 (0·3–1)	1·2 (0·6–2·1)	0·1 (0–0·1)	1·7 (0·8–3·3)
Equatorial Guinea	24·5 (20·8–27·8)	5·6 (3·6–7·9)	4·6 (2·5–7·3)	0·7 (0·3–1·3)	5·4 (2·9–8·7)
Eritrea	107 (73·7–137)	0·9 (0·5–1·5)	11·8 (5·7–21·4)	0·3 (0·1–0·6)	88·4 (55·6–124)
Eswatini	23·1 (22·1–24)	0·7 (0·4–1)	2 (1·5–2·7)	0·2 (0·1–0·2)	1·9 (1·3–2·6)
Gabon	32·8 (29–36·4)	10·9 (7·7–14·2)	1·2 (0·6–2·2)	2·3 (1·1–4·1)	1·4 (0·7–2·7)
Gambia	25·7 (21·8–29·7)	5·2 (3·6–7·2)	38·9 (34·4–43·4)	0·2 (0·1–0·3)	9·3 (6–13·6)
Ghana	520 (490–547)	87·3 (71·8–104)	162 (142–183)	5·5 (4·3–6·9)	133 (110–159)
Guinea	109 (80–140)	28·5 (18–42·6)	190 (147–232)	1·4 (0·9–2·1)	182 (127–244)
Guinea-Bissau	35·8 (27·1–44)	0·5 (0·3–0·9)	8·5 (4·7–13·6)	0·3 (0·1–0·5)	27·7 (19·2–37·9)
Kenya	571 (527–610)	172 (151–194)	220 (198–241)	7·2 (5·8–8·9)	215 (170–280)
Lesotho	28·4 (25·6–30·8)	1·9 (1·2–2·9)	7·1 (4·8–9·8)	0·1 (0–0·2)	3·8 (3–4·7)
Liberia	62·5 (51·7–73·9)	28·2 (21·2–36·4)	64 (52–75·1)	0·2 (0·2–0·3)	24·1 (15·7–34·7)
Madagascar	126 (102–151)	48·7 (36·8–61·7)	242 (203–284)	0·9 (0·7–1·3)	589 (533–647)
Malawi	369 (335–407)	20·8 (14·3–29·4)	215 (176–252)	1·9 (1·3–2·7)	15·6 (9·5–23·5)
Mali	79·2 (58·4–104)	33·2 (21·5–48·1)	663 (563–743)	13·4 (9·5–18·5)	271 (180–382)
Mauritania	63·5 (54·5–72·8)	3·8 (2·5–5·6)	42·1 (33·7–50·4)	0·1 (0·1–0·2)	28·6 (20·4–39·3)
Mauritius	9·9 (9–10·7)	1·9 (1·2–2·6)	0·5 (0·3–1)	0·2 (0·1–0·4)	0 (0–0)
Mozambique	432 (336–540)	4·8 (2·6–7·8)	341 (235–439)	2 (1·1–3·4)	427 (371–487)
Namibia	53·5 (50·1–56·2)	4·9 (3–7·3)	1·6 (0·8–2·8)	0·1 (0–0·3)	3·8 (2·1–6·2)

Niger	93 (48·1–155)	9·8 (4·1–18·9)	390 (244–565)	4·9 (2·3–9·7)	764 (551–948)
Nigeria	2241 (1910–2602)	1363 (1121–1606)	1019 (687–1361)	25·8 (15·3–41·7)	3848 (3670–4033)
Rwanda	166 (149–186)	6·1 (4·2–9)	177 (156–195)	0·6 (0·5–0·8)	15·7 (9·7–23·4)
Sao Tome and Principe	4 (3·8–4·1)	0 (0–0)	0·1 (0·1–0·2)	0 (0–0)	0·2 (0·1–0·3)
Senegal	132 (112–152)	37 (26·5–50·5)	345 (324–363)	0·6 (0·5–0·8)	42·9 (36·1–50·3)
Sierra Leone	88·9 (76·5–102)	7·6 (5·3–10·7)	132 (119–146)	0·8 (0·6–1·1)	35·5 (24·8–49·1)
Somalia	241 (146–351)	97·2 (50–155)	31·3 (13·6–61·3)	10·8 (4–23·5)	557 (399–703)
South Africa	904 (849–949)	40·3 (25·1–60·1)	84 (47·1–133)	0·7 (0·3–1·4)	30 (15·2–53·4)
South Sudan	21·1 (10·1–38·5)	2 (0·8–4)	12·3 (5·1–24)	0·3 (0·1–0·6)	277 (251–295)
Sudan	355 (231–495)	17·6 (9·2–28·9)	2·5 (1–5)	0·9 (0·3–1·8)	491 (348–622)
Togo	94·5 (75·5–116)	23·5 (14·3–35·2)	82·4 (61–102)	7 (4·2–10·6)	29·2 (17·4–46)
Tuvalu	0·2 (0·2–0·2)	0 (0–0)	0 (0–0)	0 (0–0)	0 (0–0)
Uganda	627 (511–755)	408 (307–509)	362 (254–477)	2·1 (1·2–3·4)	223 (135–338)
United Republic of Tanzania	1174 (1110–1238)	31·3 (23·4–40·7)	483 (433–536)	17·3 (13·7–22·1)	361 (305–421)
Zambia	281 (241–327)	15 (9·4–23·4)	297 (254–336)	1·1 (0·8–1·5)	68·6 (42–105)
Zimbabwe	238 (209–268)	20·5 (13·6–29·7)	139 (110–168)	1·6 (1·1–2·2)	65·3 (45·8–90·2)

Delivery location by country, 1995

Supplementary Table 6. Delivery location by country, 1995

Location	Public and private non-profit hospital	Private for-profit hospital	Public and private non-profit lower-level	Private for-profit lower-level	Non-facility
All study countries	22·1 (21·2–23)	11·3 (10·6–12·1)	6·4 (5·9–7)	0·9 (0·8–1·1)	59·2 (57·9–60·5)
Central Europe, Eastern Europe, and Central Asia	87·6 (85·8–89·2)	0·8 (0·5–1·2)	4·4 (3·5–5·6)	0·2 (0·1–0·3)	7 (5·8–8·4)
Albania	80·3 (74·1–86·1)	0·4 (0·2–0·7)	0·1 (0–0·1)	0·2 (0·1–0·4)	19 (13·2–25·2)
Armenia	18·7 (16·5–21·2)	2·1 (1·2–3·5)	72·3 (69·6–74·7)	0·1 (0·1–0·1)	6·8 (5·1–8·8)
Azerbaijan	67·9 (59·3–75·9)	0·5 (0·3–0·7)	11·2 (6·5–16·9)	1·8 (0·8–3·3)	18·6 (11·9–26·3)
Belarus	99·8 (99·7–99·9)	0 (0–0)	0·1 (0–0·1)	0·1 (0–0·1)	0 (0–0·1)
Bosnia and Herzegovina	79·1 (71·5–85·9)	0 (0–0)	15·6 (9·6–23)	0·1 (0–0·2)	5·2 (2·5–9·6)

Georgia	81·7 (77·7–85·2)	7·6 (4·9–11·2)	1·1 (0·5–2)	1·9 (1–3·5)	7·7 (6–9·6)
Kazakhstan	98·3 (97·7–98·7)	0 (0–0·1)	0·1 (0·1–0·2)	0·1 (0·1–0·2)	1·4 (1·1–2)
Kyrgyzstan	94·5 (92·8–95·8)	1·7 (1–2·8)	0·1 (0–0·2)	0·2 (0·1–0·4)	3·5 (2·4–4·9)
Mongolia	67·7 (57·4–77)	0·4 (0·2–0·7)	26·9 (17·5–37·3)	0·1 (0–0·2)	4·9 (4·4–5·5)
Montenegro	99·3 (98·9–99·5)	0·1 (0·1–0·2)	0·2 (0·1–0·4)	0·1 (0–0·2)	0·4 (0·2–0·7)
North Macedonia	59 (50·8–67·7)	2·7 (1·6–4·4)	37·5 (28·4–46)	0·3 (0·2–0·4)	0·5 (0·2–1)
Republic of Moldova	95·8 (94–97·2)	0·4 (0·2–0·6)	2·4 (1·2–3·9)	0·2 (0·1–0·4)	1·3 (0·8–1·8)
Serbia	98·5 (97·8–99)	0·5 (0·3–0·9)	0·5 (0·2–1)	0 (0–0·1)	0·4 (0·2–0·8)
Tajikistan	57·2 (52·5–62·1)	0·1 (0·1–0·2)	1·6 (0·8–2·7)	0·1 (0–0·2)	41 (36·1–45·7)
Turkmenistan	88·2 (81·6–92·8)	0·1 (0·1–0·2)	8·9 (4·7–15·2)	0·1 (0–0·3)	2·6 (1·6–3·9)
Ukraine	98·9 (98·4–99·2)	0·6 (0·3–0·9)	0·5 (0·3–0·9)	0 (0–0)	0·1 (0–0·1)
Uzbekistan	94·2 (92·1–95·8)	0·9 (0·5–1·4)	0·2 (0·1–0·3)	0 (0–0·1)	4·7 (3·1–6·7)
Latin America and Caribbean	50·2 (47·7–52·7)	14·9 (13·1–16·9)	12 (9·9–14)	1·7 (1·3–2·3)	21·2 (20·2–22·3)
Argentina	53·1 (45·1–61·5)	38·5 (30·1–47·1)	6·1 (3·1–10·4)	0·1 (0–0·2)	2·2 (1·1–3·9)
Belize	68·8 (62·3–74·3)	6·3 (5–7·9)	0 (0–0·1)	4·6 (3·4–6·1)	20·2 (14·2–27·5)
Bolivia (Plurinational State of)	25·8 (24·5–27·2)	13·4 (12·5–14·5)	7·1 (6·3–8)	0·9 (0·7–1·1)	52·8 (51·2–54·3)
Brazil	62·3 (59·8–64·7)	18·3 (15·6–21)	13·1 (12·2–14·1)	0·1 (0·1–0·1)	6·2 (5·4–7)
Colombia	55·1 (51·4–58·4)	19·9 (16·8–23·4)	6·7 (6–7·6)	0·1 (0·1–0·2)	18·1 (16·6–19·6)
Costa Rica	93·9 (91·7–95·9)	2·3 (1·3–3·8)	1·6 (0·8–3)	0·2 (0·1–0·4)	2 (1–3·5)
Cuba	73·5 (66·7–80·7)	26·2 (19–33·1)	0 (0–0)	0 (0–0·1)	0·2 (0·1–0·4)
Dominica	81·9 (76–86·9)	13·9 (8·9–19·7)	1·2 (0·5–2·3)	1 (0·4–2)	2 (0·9–3·8)
Dominican Republic	69·3 (67·8–70·6)	24·4 (23·1–25·6)	2·4 (2–2·9)	0·1 (0·1–0·1)	3·9 (3·3–4·5)
Ecuador	40·5 (34·7–46·8)	27·2 (21·3–32·7)	3·3 (1·7–5·5)	4 (2·1–6·4)	24·9 (22·8–27·4)
El Salvador	55·5 (53·5–57·1)	1 (0·6–1·6)	1·5 (0·7–2·8)	0·1 (0–0·2)	41·9 (40·5–43·3)
Grenada	78 (71·2–83·8)	16 (10·4–22·3)	1·5 (0·7–2·8)	0·7 (0·3–1·4)	3·8 (1·7–7·2)
Guatemala	28·5 (26·7–30·4)	5·7 (5–6·5)	2·4 (2–2·8)	0·5 (0·4–0·7)	62·8 (60·6–65·1)

Haiti	10·2 (8·5–11·9)	4 (2·9–5·3)	3·6 (2·8–4·4)	1·2 (0·7–1·7)	81·1 (78·7–83·2)
Honduras	44·9 (42·6–47)	2·3 (1·8–3)	2 (1·3–2·9)	1·1 (0·6–1·6)	49·7 (47·7–51·8)
Jamaica	76·4 (72·9–79·7)	6 (3·8–9)	1 (0·7–1·6)	0·4 (0·2–0·8)	16·1 (13·8–18·6)
Mexico	36 (29·3–43·6)	9·5 (5·9–14)	24 (16·9–30·8)	3·1 (1·8–4·9)	27·2 (23·6–31)
Nicaragua	55·2 (53·7–56·6)	1·2 (1–1·5)	4·4 (3·9–4·9)	2·4 (2·1–2·8)	36·8 (35·3–38·3)
Paraguay	39 (33·2–44·6)	12·6 (10·3–15)	18·5 (13·5–24·2)	1·9 (1·1–2·9)	28 (22·1–33·9)
Peru	30·4 (27·6–33)	5·5 (4·9–6·2)	7 (5·5–8·7)	9·7 (7·7–11·7)	47·5 (46·1–48·8)
Saint Lucia	96·9 (95·2–98·2)	3 (1·8–4·8)	0 (0–0)	0 (0–0)	0 (0–0)
Saint Vincent and the Grenadines	77·9 (71·4–83·7)	13·9 (8·9–19·8)	1·8 (0·8–3·2)	0·8 (0·3–1·5)	5·7 (2·7–10·9)
Suriname	42·7 (34–51·8)	25·7 (18·9–33·4)	10·4 (5·6–17·3)	0·7 (0·3–1·5)	20·5 (11·2–32·3)
Venezuela (Bolivarian Republic of)	87 (82·2–90·9)	6 (3·6–9·1)	4·1 (1·9–7·2)	2·1 (0·9–4·1)	0·8 (0·4–1·6)
North Africa and Middle East	34·3 (31·8–36·9)	10·7 (9·4–12·3)	4·7 (4·1–5·5)	1 (0·8–1·3)	49·3 (46·3–52·1)
Algeria	79·4 (74·9–83·4)	5·1 (3·4–7·1)	7·6 (4·9–10·9)	0·1 (0·1–0·2)	7·8 (5·3–11·1)
Egypt	17·7 (16·7–18·7)	18·3 (17·3–19·3)	0·7 (0·6–0·9)	0·3 (0·2–0·3)	63·1 (61·5–64·5)
Iran (Islamic Republic of)	61·9 (51·1–71·2)	17·8 (11·6–24·9)	0·4 (0·2–0·8)	0·3 (0·1–0·6)	19·5 (10·6–32·2)
Iraq	40·9 (31·1–50·8)	6·4 (4·2–9·6)	0 (0–0)	0·1 (0–0·2)	52·6 (41·2–63·7)
Jordan	56·4 (55–57·9)	36·1 (34·6–37·6)	0·3 (0·2–0·6)	0·4 (0·2–0·7)	6·7 (6–7·5)
Lebanon	8·9 (7·5–10·5)	84·4 (81·3–87)	0·6 (0·2–1·2)	3·7 (2–6·2)	2·4 (1·2–4·2)
Libya	73·1 (65·2–80·3)	23·2 (15·9–31·1)	0·9 (0·4–1·7)	0·7 (0·3–1·4)	2 (0·9–3·9)
Morocco	28·4 (24·7–32·4)	6·7 (5·4–8·2)	10·7 (8·6–12·9)	0·1 (0·1–0·1)	54·2 (48·6–59·5)
Palestine	52·3 (48·6–56)	26·6 (23·3–30·1)	0·1 (0–0·1)	14·7 (13·4–16)	6·3 (4·6–8·4)
Syrian Arab Republic	22·7 (18·8–26·8)	22 (18·2–26·1)	1·5 (0·8–2·4)	8·5 (5·9–11·7)	45·3 (39·6–51·7)
Tunisia	77·1 (70·7–82·6)	8·1 (5·9–11·1)	0 (0–0)	0 (0–0)	14·8 (9·8–21·2)
Türkiye	43·7 (41·5–45·8)	6·6 (5·6–7·8)	18·5 (17–20·1)	1·2 (1–1·4)	30 (27·9–32)
Yemen	10·4 (7·3–14·3)	2·3 (1·3–3·5)	0·7 (0·3–1·3)	0·1 (0–0·2)	86·5 (81·7–90·5)
South Asia	14·1 (13·4–14·9)	14·5 (13·7–15·3)	1·9 (1·5–2·3)	0·1 (0·1–0·1)	69·4 (68–70·6)

Afghanistan	11·1 (5·7–18·5)	3·3 (1·5–6·4)	5·7 (2·5–10·5)	1·7 (0·6–3·4)	78·2 (65·2–87·8)
Bangladesh	2·5 (2·2–2·8)	1·7 (1·5–2)	0·5 (0·4–0·6)	0·5 (0·4–0·6)	94·8 (94·4–95·4)
Bhutan	10·1 (5·2–16·4)	6·9 (3·5–11·7)	5·5 (2·2–10)	1·5 (0·5–3·1)	76·1 (63·7–86·7)
India	15 (14·2–15·8)	15·2 (14·4–16·2)	2·1 (1·7–2·5)	0·1 (0–0·1)	67·7 (66·1–69·1)
Nepal	5·6 (4·9–6·5)	1·1 (0·8–1·5)	0·8 (0·5–1)	0·2 (0·1–0·3)	92·4 (91·4–93·3)
Pakistan	9·4 (7·7–11·2)	12·1 (10·1–14·3)	0·4 (0·2–0·7)	0·1 (0·1–0·2)	78 (74·6–81·4)
Southeast Asia, East Asia, and Oceania	25·4 (24·3–26·6)	6·2 (5·8–6·6)	7·5 (6·6–8·6)	4·5 (4–5)	56·4 (54·8–57·9)
Cambodia	6·5 (5·2–8)	0·6 (0·4–0·9)	1·9 (1·3–2·6)	1·9 (1·4–2·7)	89 (86·7–91)
Democratic People's Republic of Korea	67·7 (58·8–75·9)	11·4 (7·1–16·9)	16·7 (9·6–25·5)	2·6 (1·1–5·1)	1·6 (1·1–2·5)
Fiji	90·7 (86·1–94·1)	0 (0–0)	5·8 (2·9–9·9)	0·1 (0–0·2)	3·4 (1·7–6·2)
Indonesia	7·5 (6·7–8·5)	6 (5·3–6·7)	2·6 (2·2–3)	9·6 (8·6–10·7)	74·3 (71·8–76·7)
Kiribati	49·3 (37·6–60·8)	0 (0–0)	21·8 (13·3–31·7)	0·1 (0–0·2)	28·8 (17·5–42·5)
Lao People's Democratic Republic	9·7 (5·8–15)	0 (0–0)	5 (2·5–8·5)	0·7 (0·3–1·4)	84·6 (77·7–90)
Malaysia	65·9 (56·4–74·9)	4·1 (2·3–6·5)	16·2 (9·4–24)	6·9 (3·4–11·6)	7 (4–11)
Maldives	49·1 (37–60·3)	8·2 (4·9–12·9)	10·4 (5·4–17·7)	0·1 (0–0·3)	32·2 (19–47·5)
Marshall Islands	66·4 (56·6–75·2)	1·6 (0·9–2·5)	15·4 (8·6–23·2)	0·5 (0·2–1·1)	16·1 (9·1–25·6)
Micronesia (Federated States of)	57·7 (45·1–68·9)	0·4 (0·2–0·8)	15·9 (8·9–23·7)	0·7 (0·3–1·4)	25·3 (14·2–39·6)
Myanmar	10 (6·1–15·6)	3·4 (1·7–5·9)	2·7 (1·2–5)	0·1 (0–0·3)	83·7 (75·3–89·8)
Papua New Guinea	22·7 (16·7–29·6)	1·1 (0·6–1·9)	26·5 (19·5–33·2)	0·1 (0–0·2)	49·5 (47·2–51·8)
Philippines	17·7 (16·7–18·8)	13·5 (12·6–14·4)	1·5 (1·3–1·9)	0·1 (0·1–0·2)	67·1 (65·6–68·5)
Samoa	69·1 (58·4–78·6)	0 (0–0)	12 (6·4–19·4)	0·1 (0–0·2)	18·7 (10·4–30·2)
Solomon Islands	52 (41·1–63·4)	0·6 (0·4–1·1)	26·1 (16·1–36·4)	0·4 (0·2–0·8)	20·8 (12·2–32·3)
Sri Lanka	91·2 (89–93·1)	0·2 (0·1–0·3)	0 (0–0)	6·5 (4·8–8·4)	2·1 (1·4–3)
Thailand	81·5 (76–86·2)	7·6 (4·9–10·9)	0·4 (0·2–0·9)	3·8 (2–6·3)	6·7 (3·6–11·2)
Timor-Leste	3·7 (2·2–5·7)	0·1 (0–0·1)	1·6 (0·9–2·7)	0·2 (0·1–0·4)	94·4 (91·7–96·3)
Tonga	91·9 (87·3–95·3)	0·5 (0·3–0·8)	0·1 (0–0·2)	1 (0·5–1·8)	6·5 (3·2–11·4)

Vanuatu	52·1 (41·1–63·5)	0·5 (0·3–0·9)	26·3 (16–36·6)	0·5 (0·2–0·9)	20·6 (11·7–32·8)
Viet Nam	26·9 (23·9–30)	1·4 (1–1·8)	30·9 (27·6–34·3)	0·2 (0·2–0·3)	40·6 (35·5–45·8)
Sub-Saharan Africa	16 (14·7–17·4)	7 (6·1–8·1)	13·8 (12·6–15·1)	0·9 (0·6–1·2)	62·3 (60·2–64·4)
Angola	12·6 (7·2–19·9)	0·3 (0·1–0·7)	11·3 (6·2–17·6)	0·1 (0–0·1)	75·7 (64·1–84·7)
Benin	10·6 (9·6–11·7)	6·3 (5·4–7·2)	48·5 (46·3–50·6)	0·1 (0·1–0·1)	34·6 (32–37·1)
Botswana	73·5 (64·9–80·7)	3·4 (1·9–5·4)	13·5 (7·5–20·7)	0·2 (0·1–0·4)	9·4 (5–15·9)
Burkina Faso	2 (1·7–2·3)	0·6 (0·4–0·7)	29·5 (27·5–31·7)	0·2 (0·2–0·3)	67·7 (65·3–69·9)
Burundi	11·3 (6·1–18·4)	1·2 (0·5–2·3)	17·7 (10·1–26·8)	5·4 (2·6–9·2)	64·4 (48·3–78)
Cabo Verde	26·2 (17·8–36·3)	5 (2·6–8·4)	12·8 (7–20·3)	0·1 (0·1–0·3)	56 (42·2–68·6)
Cameroon	24·7 (22–27·5)	4·4 (3·3–5·7)	26·2 (23·3–29)	0·1 (0·1–0·2)	44·5 (40·4–48·6)
Central African Republic	19·1 (16·4–22·1)	1·6 (1·1–2·3)	27 (23·7–30·3)	0·9 (0·7–1·2)	51·3 (46·9–56)
Chad	7·2 (6·4–8)	0·6 (0·4–0·8)	3·5 (3–4·1)	0·5 (0·4–0·7)	88·2 (87–89·3)
Comoros	37 (33·6–40·3)	0·1 (0·1–0·2)	6·1 (4·6–8)	0·1 (0·1–0·2)	56·7 (53·4–60·2)
Congo	60·4 (52–67·6)	2·1 (1·1–3·4)	8·6 (5–13·2)	7·6 (6·1–9·2)	21·3 (13–31·3)
Côte d'Ivoire	6·8 (6–7·7)	0·9 (0·6–1·3)	31·3 (29–33·8)	0·2 (0·2–0·3)	60·7 (57·7–63·4)
Democratic Republic of the Congo	15 (10–21·1)	11·3 (7·4–16·2)	36·8 (27·5–45·6)	9·1 (5·6–13·1)	27·9 (16·8–42)
Djibouti	58·5 (48·5–67·4)	1·3 (0·8–2)	10 (6·3–14·9)	0·1 (0·1–0·2)	30·2 (20·1–40·6)
Equatorial Guinea	2·9 (1·3–5·2)	0·4 (0·2–0·8)	2·8 (1·2–5·2)	0·2 (0·1–0·4)	93·8 (89·1–97)
Eritrea	12·9 (6·8–21·1)	0·1 (0·1–0·2)	1·8 (0·9–3·2)	0·1 (0–0·1)	85·1 (75·3–92·1)
Eswatini	57·4 (47·6–66·8)	5·2 (3·1–7·8)	6·9 (3·7–11·7)	0·3 (0·1–0·6)	30·2 (20·4–42·6)
Gabon	53·8 (44·6–61·9)	13·9 (10·7–17)	6·7 (3·3–11·4)	8·4 (4·2–14·6)	17·1 (9·4–28·5)
Gambia	9·5 (5·5–14·7)	2 (0·9–3·6)	35·6 (23·9–47·7)	0·4 (0·2–0·6)	52·6 (37·8–68)
Ghana	29·2 (26·9–31·3)	5·8 (4·6–7·2)	9·4 (7·9–11·1)	0·4 (0·3–0·6)	55·1 (53·4–56·7)
Guinea	7 (5·9–8·1)	1·7 (1·1–2·6)	19·6 (17·9–21·3)	0·1 (0·1–0·2)	71·5 (69·5–73·5)
Guinea-Bissau	20·3 (11·4–31·5)	0·5 (0·2–0·9)	8·7 (4–15·4)	0·2 (0·1–0·4)	70·3 (54·9–82·7)
Kenya	36·7 (34·4–38·9)	3·5 (2·9–4·3)	6·1 (5–7·2)	0·2 (0·1–0·3)	53·5 (51·1–55·8)

Lesotho	39·2 (31·5–47)	1·3 (0·8–2·1)	7·4 (4·3–11·4)	0·1 (0·1–0·2)	51·9 (42·6–61)
Liberia	7·1 (3·8–11·6)	4·6 (2·3–7·9)	17 (10–25·9)	0·1 (0–0·2)	71·2 (58·3–82·1)
Madagascar	25·2 (23·1–27·5)	1·3 (1–1·6)	9·6 (8·3–11·1)	0·1 (0·1–0·1)	63·9 (61·2–66·4)
Malawi	27·1 (24·9–29·2)	1·8 (1·4–2·4)	26·8 (24·8–28·9)	0·1 (0·1–0·2)	44·1 (41·1–47·1)
Mali	3·4 (2·9–3·9)	0·6 (0·5–0·8)	32·4 (30·6–34·5)	0·4 (0·3–0·5)	63·1 (60·8–65·2)
Mauritania	27·1 (20·1–33·7)	2·1 (1·4–3)	25·1 (18·9–31·3)	0·1 (0·1–0·2)	45·6 (33·7–58·6)
Mauritius	77·3 (70·2–83·5)	11 (6·8–16·2)	7 (3·5–11·9)	3·1 (1·3–6)	1·6 (0·9–2·6)
Mozambique	9·4 (6·9–12·8)	0·1 (0·1–0·2)	22 (17·8–26·6)	0·1 (0·1–0·1)	68·4 (62·6–73·5)
Namibia	66·3 (63·1–69·4)	3·7 (2·9–4·7)	3·4 (2·7–4·4)	0·1 (0·1–0·2)	26·4 (23·4–29·8)
Niger	0·8 (0·6–1)	0·2 (0·2–0·4)	16·3 (14·4–18·3)	0·2 (0·1–0·2)	82·6 (80·3–84·6)
Nigeria	11 (8·8–13·3)	12·5 (10·3–14·7)	6·5 (4·6–8·5)	0·2 (0·1–0·3)	69·9 (67·6–72)
Rwanda	11·6 (10·3–13)	0·8 (0·6–1·1)	12·8 (11·4–14·2)	0·1 (0·1–0·1)	74·7 (72·3–76·7)
Sao Tome and Principe	44 (33·4–55·4)	0 (0–0)	8·5 (4·5–14·2)	0·1 (0–0·1)	47·3 (34·6–60)
Senegal	11·6 (10·6–12·7)	3·4 (2·9–4)	32·2 (30·6–33·8)	0·1 (0·1–0·1)	52·7 (50·8–54·7)
Sierra Leone	5·9 (3–9·9)	0·8 (0·3–1·7)	23·8 (14·4–35·2)	0·2 (0·1–0·4)	69·3 (55·1–81·5)
Somalia	5·5 (2·8–9·5)	1·4 (0·6–2·6)	1·4 (0·5–2·8)	0·6 (0·2–1·4)	91·2 (84·8–95·6)
South Africa	69·9 (68·6–71·1)	8·5 (7·5–9·6)	9·5 (9–10·1)	0·1 (0–0·1)	12 (11·4–12·6)
South Sudan	7·3 (3·5–12·5)	0·7 (0·3–1·4)	2·1 (0·7–4·3)	0 (0–0·1)	89·8 (82·7–95)
Sudan	4·9 (2·4–8·7)	0·5 (0·2–1)	0·5 (0·2–1·1)	0 (0–0·1)	94·1 (89·3–97·1)
Togo	12·8 (10·8–15)	3·5 (2·6–4·5)	32·6 (29–36·5)	0·8 (0·6–1)	50·3 (45·3–55·1)
Tuvalu	98·3 (97·5–98·9)	1·2 (0·7–1·9)	0·2 (0·1–0·3)	0·3 (0·1–0·6)	0·1 (0–0·2)
Uganda	13·1 (12–14·2)	14·6 (13·6–15·8)	7·7 (6·9–8·6)	0·1 (0·1–0·1)	64·5 (62·5–66·5)
United Republic of Tanzania	24·6 (23·1–25·9)	1·7 (1·4–2·2)	17·7 (16·6–18·8)	0·5 (0·4–0·6)	55·5 (53·9–57·1)
Zambia	20·1 (18·9–21·4)	5 (4·4–5·8)	20·2 (18·9–21·5)	0·1 (0·1–0·1)	54·6 (52·7–56·4)
Zimbabwe	39·7 (37·9–41·5)	3·2 (2·5–4)	29 (27·4–30·8)	0·2 (0·1–0·2)	27·9 (25·9–30)

Change in delivery location by country, 1995-2023

Supplementary Figure 19. Change in public and private non-profit hospital delivery, 1995-2023

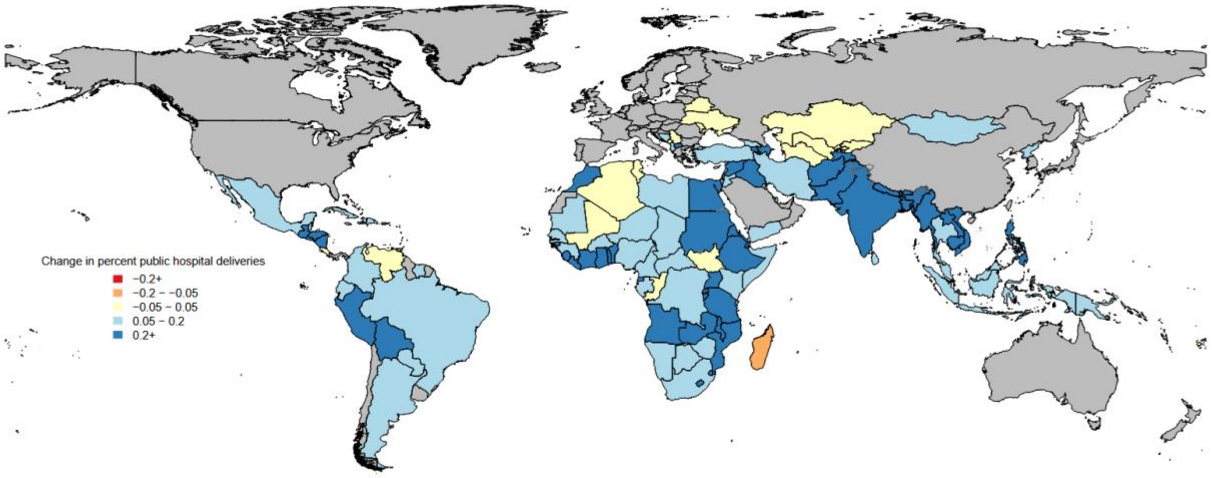

Supplementary Figure 20. Change in private for-profit hospital delivery, 1995-2023

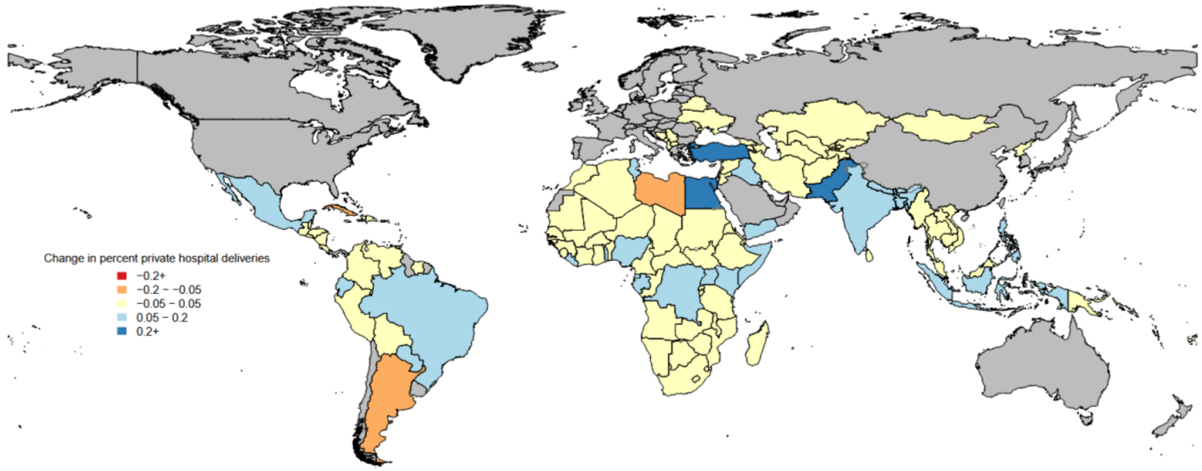

Supplementary Figure 21. Change in public and non-profit lower-level delivery, 1995-2023

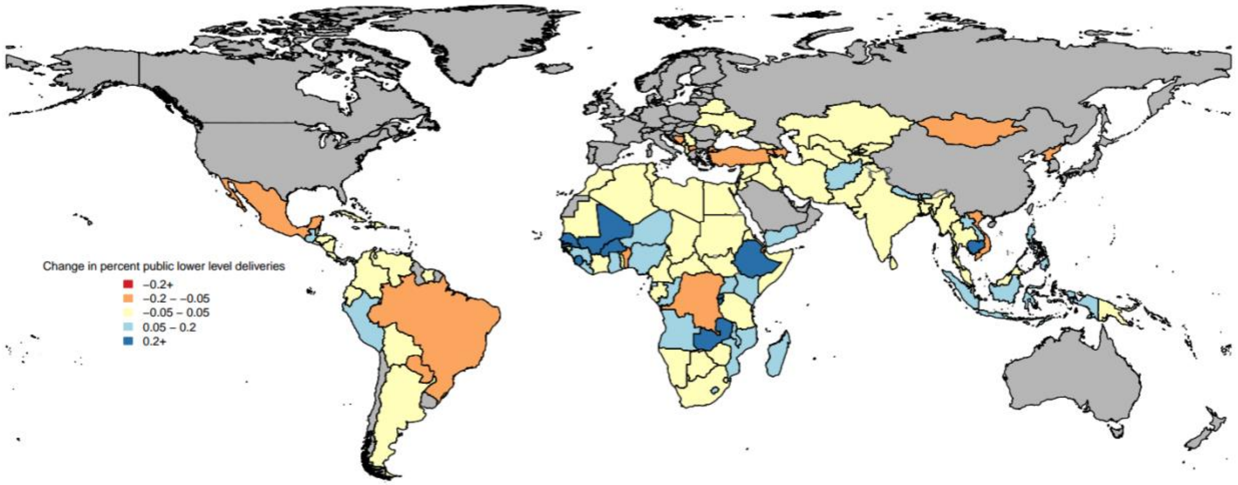

Supplementary Figure 22. Change in private lower-level delivery, 1995-2023

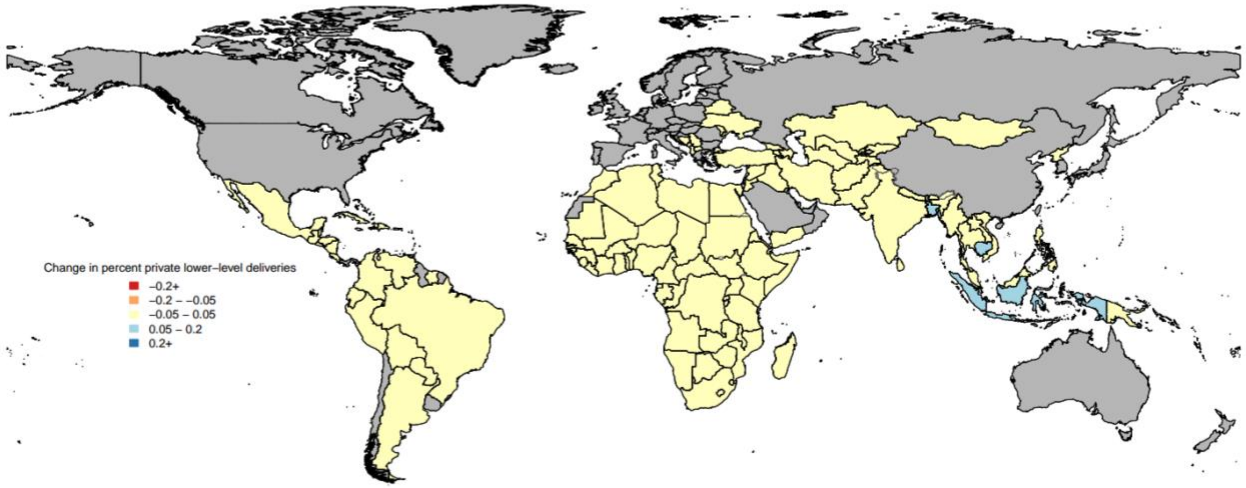

Delivery location mix versus sociodemographic index and neonatal mortality by region, 2023

Supplementary Figure 23. Facility delivery location mix versus SDI by region, 2023

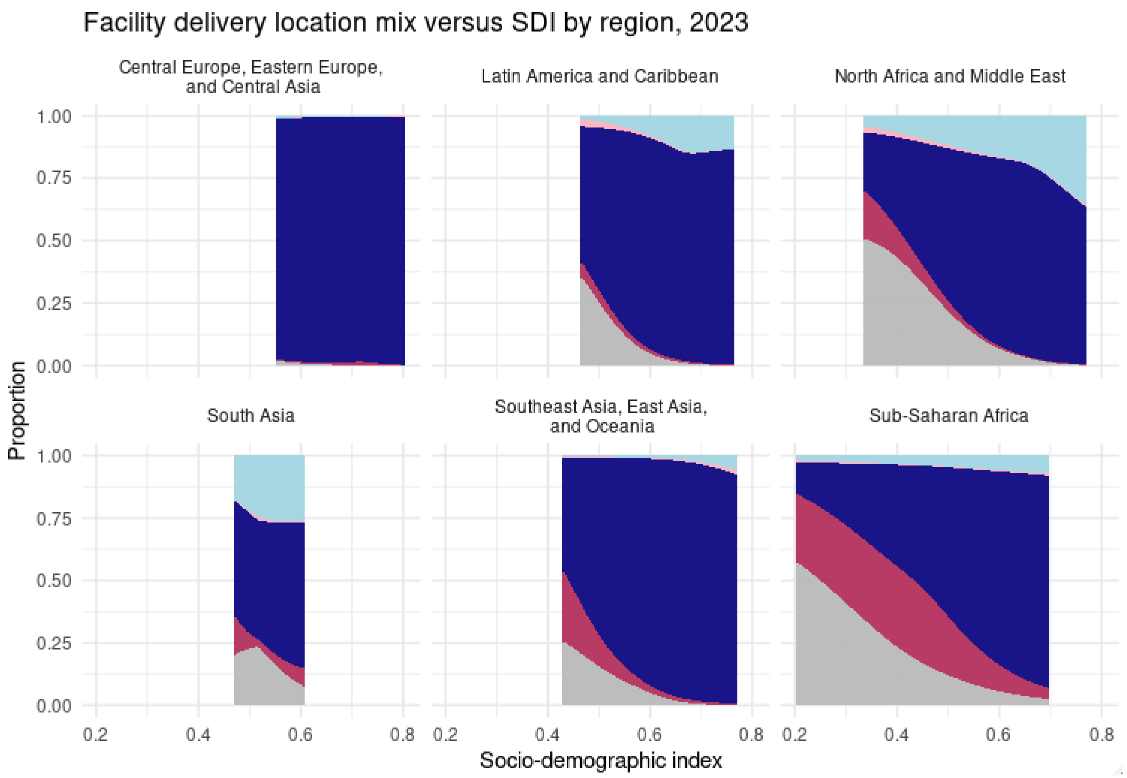

Notes: SDI: Socio-demographic index. Each region is plotted for the observed range of SDI values in the region

Supplementary Figure 24. Facility delivery location mix versus neonatal mortality by region, 2023

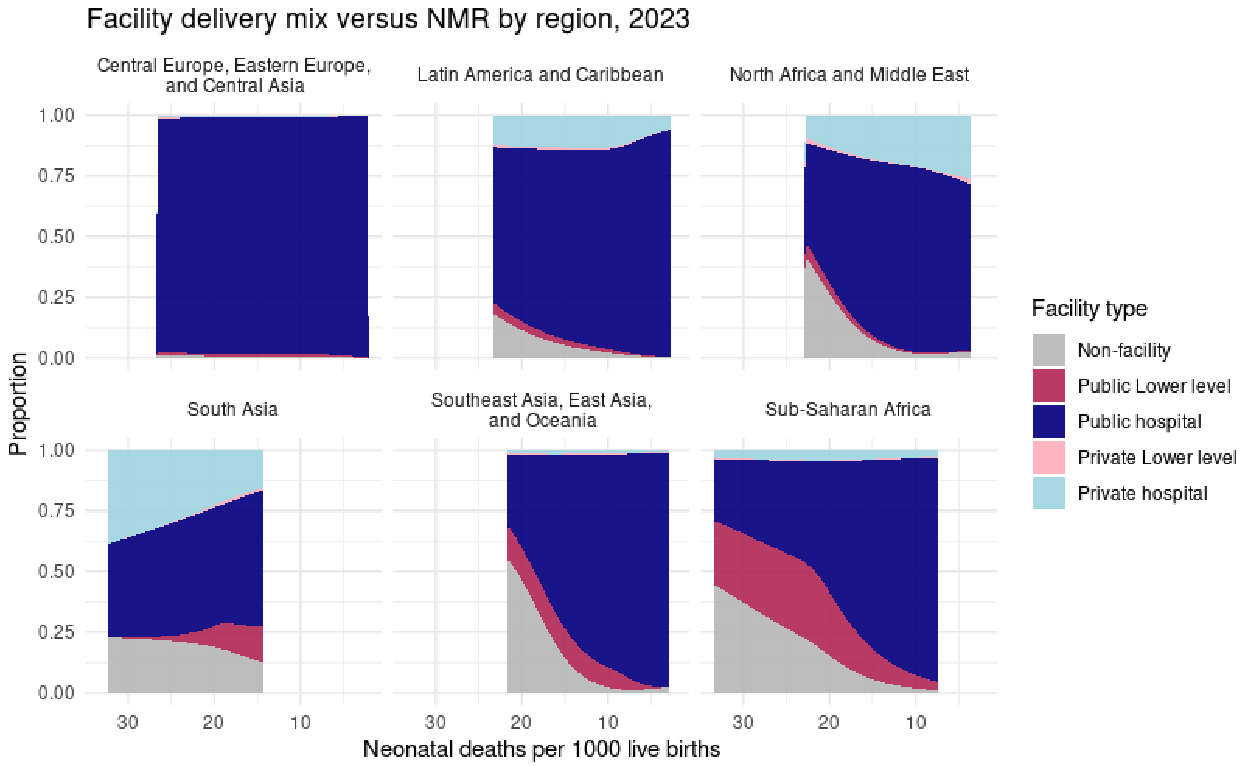

Notes: NMR: Neonatal mortality rate. Each region is plotted for the observed range of neonatal death values in the region

Delivery location mix versus maternal health, 2023

Supplementary Figure 25. Delivery location mix versus maternal mortality rate deaths, 2023

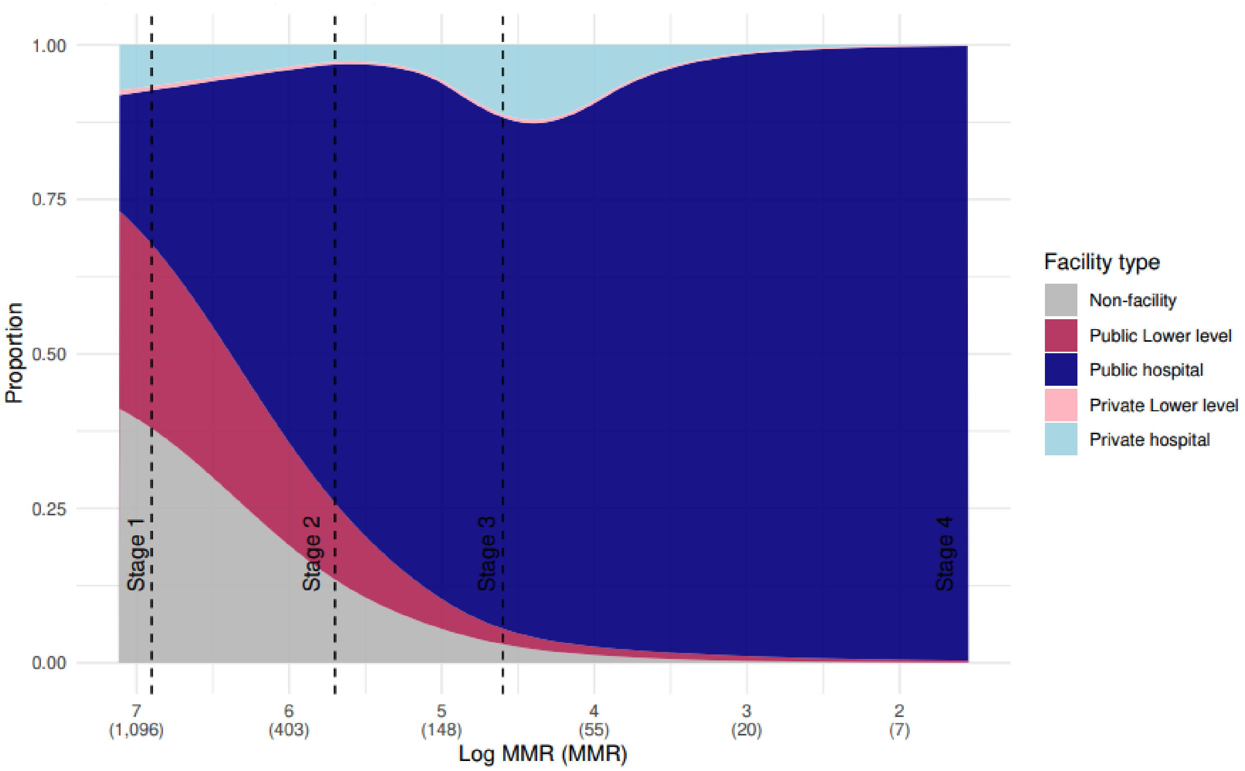

Notes: MMR: Maternal mortality ratio. MMR is grouped into stages of the obstetric transition20 and has a reverse scale. MMR sourced from the Global Burden of Diseases, Risk Factors, and Injuries Study 2023.

References

1 Stevens GA, Alkema L, Black RE, *et al.* Guidelines for accurate and transparent health estimates reporting: the GATHER statement. *The Lancet* 2016; **388**: e19–23.

2 Zimbabwe Demographic and Health Survey 2015 Final Report. Harare, Zimbabwe: Zimbabwe National Statistics Agency and The DHS Program, ICF International, 2016 https://dhsprogram.com/pubs/pdf/FR322/FR322.pdf (accessed May 29, 2025).

3 Gage AD, Fink G, Ataguba JE, Kruk ME. Hospital delivery and neonatal mortality in 37 countries in sub-Saharan Africa and South Asia: An ecological study. *PLOS Medicine* 2021; **18**: e1003843.

4 Herrera CA, Rada G, Kuhn-Barrientos L, Barrios X. Does Ownership Matter? An Overview of Systematic Reviews of the Performance of Private For-Profit, Private Not-For-Profit and Public Healthcare Providers. *PLOS ONE* 2014; **9**: e93456.

5 Global burden and 88 risk factors and subnational locations, 1990: a systematic analysis for the Global Burden of Disease Study *The Lancet* https://www.thelancet.com/journals/lancet/article/PIIS0140-6736(24)00933-4/fulltext (accessed May 13, 2025).

6 Murray CJ, Ezzati M, Flaxman AD, *et al.* GBD 2010: design, definitions, and metrics. *The Lancet* 2012; **380**: 2063–6.

7 Metreau E, Young KE, Eapen SG. World Bank country classifications by income level for 2024-2025. World Bank Blogs. 2024; published online July 1. https://blogs.worldbank.org/en/opendata/world-bank-country-classifications-by-income-level-for-2024-2025 (accessed May 13, 2025).

8 Schumacher AE, Kyu HH, Aali A, *et al.* Global age-sex-specific mortality, life expectancy, and population estimates in 204 countries and territories and 811 subnational locations, 1950–2021, and the impact of the COVID-19 pandemic: a comprehensive demographic analysis for the Global Burden of Disease Study 2021. *The Lancet* 2024; **403**: 1989–2056.

9 Haakenstad A, Yearwood JA, Fullman N, *et al.* Assessing performance of the Healthcare Access and Quality Index, overall and by select age groups, for 204 countries and territories, 1990–2019: a systematic analysis from the Global Burden of Disease Study 2019. *The Lancet global health* 2022; **10**: e1715–43.

10 Institute For Health Metrics And Evaluation. Global Burden of Disease Study 2021 (GBD 2021) Covariates 1980-2021. 2024. DOI:10.6069/B09T-1R53.

11 Bhattacharjee NV, Schumacher AE, Aali A, *et al.* Global fertility in 204 countries and territories, 1950–2021, with forecasts to 2100: a comprehensive demographic analysis for the Global Burden of Disease Study 2021. *The Lancet* 2024; **403**: 2057–99.

12 Lozano R, Fullman N, Mumford JE, *et al.* Measuring universal health coverage based on an index of effective coverage of health services in 204 countries and territories, 1990–2019: a systematic analysis for the Global Burden of Disease Study 2019. *The Lancet* 2020; **396**: 1250–84.

13 Zheng P, Barber ,Ryan, Sorensen ,Reed J. D., Murray ,Christopher J. L., and Aravkin AY. Trimmed Constrained Mixed Effects Models: Formulations and Algorithms. *Journal of Computational and Graphical Statistics* 2021; **30**: 544–56.

14 Naghavi M, Ong KL, Aali A, *et al.* Global burden of 288 causes of death and life expectancy decomposition in 204 countries and territories and 811 subnational locations, 1990–2021: a systematic analysis for the Global Burden of Disease Study 2021. *The Lancet* 2024; **403**: 2100–32.
